# The genomes of Australian wild limes

**DOI:** 10.1007/s11103-024-01502-4

**Published:** 2024-09-24

**Authors:** Upuli Nakandala, Agnelo Furtado, Ardashir Kharabian Masouleh, Malcolm W. Smith, Patrick Mason, Darren C. Williams, Robert J. Henry

**Affiliations:** 1 Queensland Alliance for Agriculture and Food Innovation, University of Queensland, Brisbane, 4072 Australia; 2 ARC Centre of Excellence for Plant Success in Nature and Agriculture, University of Queensland, Brisbane, 4072 Australia; 3 Department of Agriculture and Fisheries, Bundaberg Research Station, Bundaberg, QLD 4670 Australia; 4 Herbalistics Pty Ltd, Bli Bli, QLD 4560 Australia

**Keywords:** Australian wild limes, Chromosome level genomes, Collinearity, Species-specific gene families, Expanded gene families, Biotic and abiotic stresses

## Abstract

Australian wild limes occur in highly diverse range of environments and are a unique genetic resource within the genus *Citrus*. Here we compare the haplotype-resolved genome assemblies of six Australian native limes, including four new assemblies generated using PacBio HiFi and Hi-C sequencing data. The size of the genomes was between 315 and 391 Mb with contig N50s from 29.5 to 35 Mb. Gene completeness of the assemblies was estimated to be from 98.4 to 99.3% and the annotations from 97.7 to 98.9% based upon BUSCO, confirming the high contiguity and completeness of the assembled genomes. High collinearity was observed among the genomes and the two haplotype assemblies for each species. Gene duplication and evolutionary analysis demonstrated that the Australian citrus have undergone only one ancient whole-genome triplication event during evolution. The highest number of species-specific and expanded gene families were found in *C. glauca* and they were primarily enriched in purine, thiamine metabolism, amino acids and aromatic amino acids metabolism which might help *C. glauca* to mitigate drought, salinity, and pathogen attacks in the drier environments in which this species is found. Unique genes related to terpene biosynthesis, glutathione metabolism, and toll-like receptors in *C. australasica*, and starch and sucrose metabolism genes in both *C. australis* and *C. australasica* might be important candidate genes for HLB tolerance in these species. Expanded gene families were not lineage specific, however, a greater number of genes related to plant-pathogen interactions, predominantly disease resistant protein, was found in *C. australasica* and *C. australis*.

**Supplementary Information:**

The online version contains supplementary material available at 10.1007/s11103-024-01502-4.

## Key message

The haplotype-resolved genomes for six Australian native taxa have enabled the identification of their novel gene repertoire and characterization of genes related to plant pathogens and abiotic stress responses.

**Supplementary Information:**

The online version contains supplementary material available at 10.1007/s11103-024-01502-4.

## Introduction

*Citrus* occur naturally in certain tropical and sub-tropical regions and are now widely cultivated around the world due to their high economic and nutritional value (Shi et al. 2023; Wu et al. 2014). Australian *Citrus* are adapted to xerophytic and rainforest climatic conditions, and are an important genetic resource, as they have many beneficial traits, including tolerance to drought, salt and diseases, shortened fruiting period, and high phenotypic diversity. There are six species endemic to Australia (Delort and Yuan 2018). *Citrus glauca* (Lindlay) Burkill is endemic to semi-arid regions of Queensland, New South Wales, and South Australia. Other native *Citrus* including *C. australasica* F. Muell, *C. australis* Planch., *C. inodora* F.M. Bailey, and *C. garrawayi* F. M. Bailey are endemic to rainforest habitats with their geographical distributions extending from Cape York peninsula to Northern New South Wales (Bani Hashemian et al. 2010). *C. gracilis* Mabb. is endemic to the Northern Territory (Mabberley 1998). *C. australasica* and *C. glauca* have attracted significant attention for commercial fruit production targeting both local and international markets (Ashmore 2014).

**Fig. 1:**
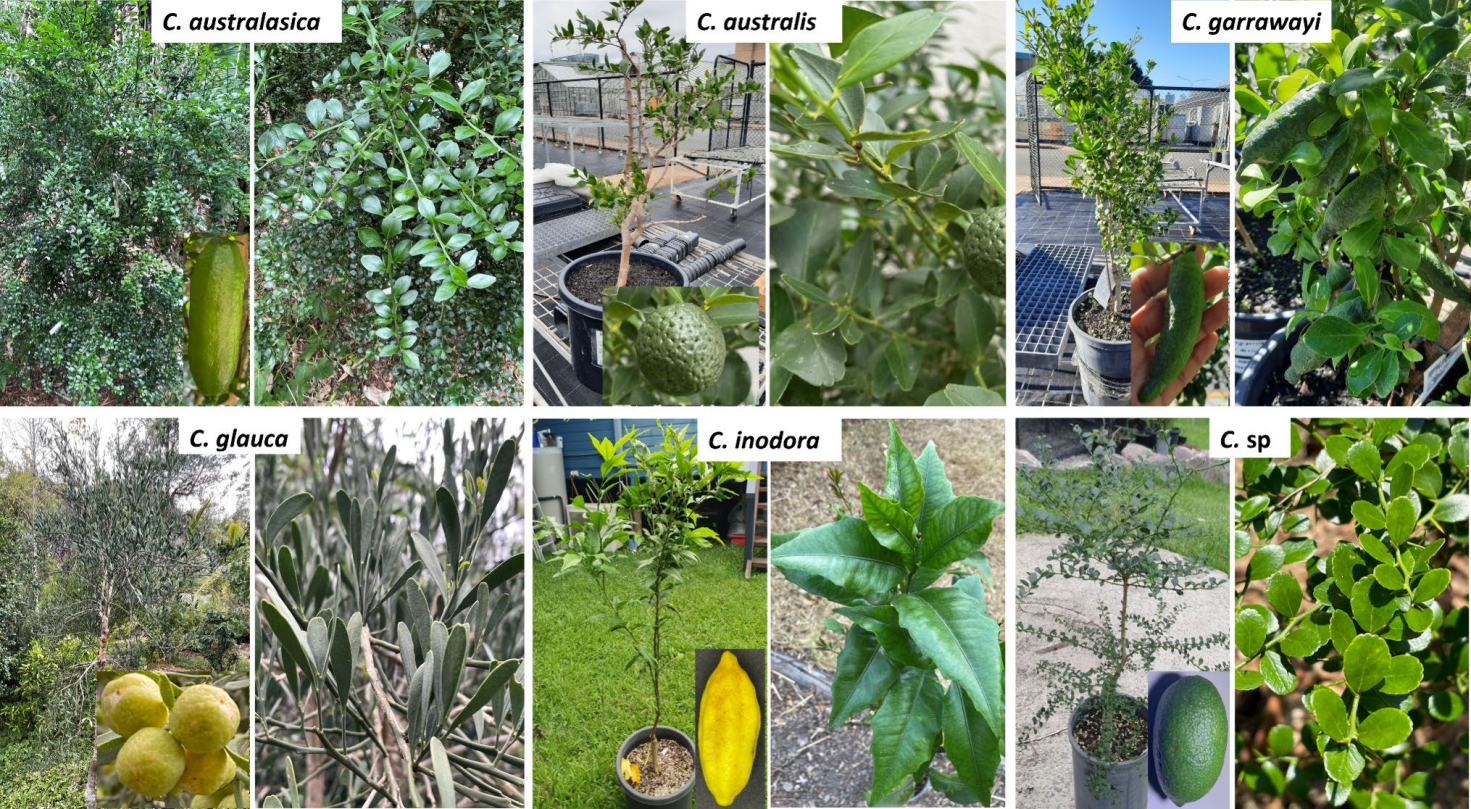
Tree structure, fruits, and foliage of the six Australian native citrus taxa used in this study

Australian limes are important genetic resources with potential for use in *Citrus* improvement (Fig. 1). *C. glauca* is a xerophyte, and has the most pronounced adaptations to survive in extreme drought conditions of any member in the *Citrus* family (Douglas 2017). Remarkably, this species can also survive in freezing temperatures (Ramadugu et al. 2017; Swingle and Reece 1967), and its hybridization with other species has shown the potential to transmit this cold hardiness to other citrus (Yelenosky et al. 1978). Its drought tolerance and other important characteristics, such as low susceptibility to boron toxicity, cold and salt tolerance, nematode resistance and graft compatibility with other *Citru*s has made this species a potential rootstock for cultivated *Citrus* species (Ashmore 2014; Scora and Ahmed 1995). *C. glauca* and *C. australis* have also been identified as poor hosts to viroids (Bani Hashemian et al. 2010). Most importantly, *C. australasica*, *C. australis*, *C. glauca* and *C. inodora* have shown different levels of resistance to Huanglongbing (HLB) disease, which has become a significant threat to *Citrus* cultivation. *C. australasica* is the best known of the Australian limes, with its high natural phenotypic diversity, making it most attractive in domestic and international markets (Rennie 2017). Furthermore, several studies have explored the large diversity of volatiles of these species (Brophy et al. 2001), which could be related to their improved resistance to pathogens (Killiny et al. 2018). These factors are indicative of Australian *Citrus*’ wide potential to be used in breeding new scion and rootstock cultivars as well as ornamentals.

Recent advances in genomic sequencing technologies have led to the development of chromosome-level genomes for some *Citrus* species, including *C. sinensis* (Gao et al. 2023; Wang et al. 2021), *C. limon* (Bao et al. 2023), *C. maxima* (Zheng et al. 2023) and relatives of *Citrus* such as *Poncirus trifoliata* (Peng et al. 2020), *Murraya paniculata* (Yang et al. 2023) and *Atalantia buxifolia* (Yuan et al. 2019). High-quality, gap free genomes for *Citrus* have been developed using Pacific Biosciences circular consensus sequencing (CCS), high-throughput chromatin capture (Hi-C), and Oxford Nanopore Technology (ONT), which have led to the identification of candidate genes associated with important characteristics in *Citrus* (Bao et al. 2023). High-quality reference genomes and chromosome-level annotation data are available for two of the Australian *Citrus* species, *C. australis*, and *C. australasica* (Nakandala et al. 2023b, 2024). Here, we generated, and characterized four additional chromosome level genome sequences for *C. garrawayi*, *C. glauca*, *C. inodora* and another distinct form of *C. garrawayi* (*C*. sp). The main aim of this study was to understand the chromosomal structure, heterozygosity, gene and repeat compositions, gene functions, different modes of structural rearrangements, and important gene families of all the Eastern Australian native taxa for the first time. The structure and the composition of these newly assembled genomes were also compared with *C. australis*, *C. australasica* and other citrus and relatives for the first time to understand the Citrus evolution and useful genes as a resource for future research and Citrus breeding.

## Materials and methods

### Plant materials and sequencing

Young, immature leaf samples of six Australian native *Citrus* species (*C. australasica* cv. Rainbow, *C. australis*, *C. garrawayi, C. glauca, C. inodora*, and another *C*. sp (a distinct form of *C. garrawayi*) were collected as described by (Nakandala et al. 2023a). Pulverized leaf tissues were used to extract DNA using a CTAB (Cetyltrimethyl ammonium bromide) DNA extraction protocol (Furtado 2014a) and to extract RNA using Trizol and Qiagen kit methods (Furtado 2014b). The PacBio circular consensus sequencing (CCS) (using PacBio Sequel II) and RNA-sequencing (with paired end read length is 150 bp) were performed at The Australian Genome Research Facility (AGRF), The University of Queensland, Australia. The Illumina sequencing (with paired end read length is 150 bp) of *C. australis*, *C. garrawayi*, *C. glauca*, *C.* sp., was conducted at the Ramaciotti Centre, University of New South Wales, NSW, Australia, and *C. australasica* [five cultivars mentioned as in (Nakandala et al. 2023a)], and *C. inodora* species were sequenced at Australian Genome Research Facility (AGRF), Melbourne, Australia.

### Genome assembly, annotation, repeat and collinearity analysis

Genome assembly and structural annotation were performed as described earlier (Nakandala et al. 2024). The functional annotation was also performed as mentioned earlier (Nakandala et al. 2023b). Repeat analysis was performed for all the species including four additional publicly available genomes (Table S1). The statistical correlation between the genome size and the repeat content (%) of the Australian limes was evaluated using IBM SPSS 25 package. Prior to the evaluation, normality of the data distribution was assessed using the Shapiro–Wilk test and Skewness/kurtosis evaluations. The data set was found to be normally distributed (Fig. S1a). Therefore, a Pearson correlation was conducted as a parametric test to evaluate the correlation. The significance level was set at p < 0.05.

Inter-genome and intra-genome collinearity blocks were identified using MCScanX. The collinear blocks for all pairs of chromosomes were computed by whole-genome BLASTP with an e value of 1e−10. The origins of gene duplications were classified using duplicate gene classifier module incorporated in MCScanX. (Wang et al. 2012). The collinear links identified by MCScanX were plotted on chromosomes using the web tool; SynVisio (https://synvisio.github.io/).

### K-mer analysis

Raw Illumina paired-end reads were trimmed at 0.01 quality limit in CLC Genomics Workbench v23,0.4 (Qiagen, USA). The raw reads of three non-Australian wild *Citrus* species were obtained from SRA database, NCBI (https://www.ncbi.nlm.nih.gov/sra) (Table S1). K-mer analysis was conducted using 0.01 quality trimmed reads using Jellyfish (Manekar and Sathe 2018), using 21 as the k-mer length. K-mer histograms were visualized using genomescope (Vurture et al. 2017) (http://qb.cshl.edu/genomescope/).

### Whole genome duplication (WGD) analysis

WGD and divergence events between species were explored based on ks peaks in WGDI (Sun et al. 2022). The details of the non-Australian species used for this analysis are indicated in Table S1. The protein alignments were performed using Diamond with an e value = 1e−5, and outfmt = 6. Collinearity analysis was performed using an algorithm in WGDI. Orthologous or paralogous protein sequences were aligned using MUSCLE software. The k_a_ and k_s_ substitution rates for each gene pair identified by collinear algorithm were estimated using the Nei-Gojobori method in PAML. The gene pairs belonging to collinear blocks and the median and average k_s_ values of collinear blocks were estimated using wgdi (-bi) program with default parameters. Ks peaks were estimated with tandem false option and other standard parameters using wgdi (-kp) program. Gaussian fitting of ks distribution was performed using PeaksFit (-pf) program. Ks distributions obtained by Peaksfitting were displayed through wgdi -kf program. The divergence time was calculated using the formula T (divergence time) = Ks/r (1 ~ 2 × 1e−9).

### Conserved and unique gene family analysis

Unique and shared orthologous gene families were identified using Orthtofinder algorithm in Orthovenn3 (Sun et al. 2023). The details of the non-Australian species used for this analysis are indicated in Table S1. All vs all protein similarities among the longest protein isoform of each species were performed with an e value cut off of 1e−5, to identify the most similar sequence for each protein sequence. Then the orthofinder clustering algorithm was used to cluster a group of sequences (gene families) which are more similar in sequence. An inflation value of 1.5 was used to generate the orthologous clusters.

### Gene family expansion and contraction analysis

The expanded and contracted gene families were identified using CAFE5 module incorporated in Orthovenn3 using the longest protein isoform of each species (Sun et al. 2023). In CAFE5, the number of genes for each gene family for each species, identified using the above step was first tabulated and filtered to remove the gene families with ≥ 100 gene copies. The species tree was generated using single copy genes. The phylogenetic tree was performed by maximum likelihood method using FastTree2 program with JTT + CAT as the evolutionary model. The birth–death (λ) parameter for the tree and the gene family counts obtained from the previous steps, was estimated within CAFÉ. The λ parameter is an indication of the probability of the gene gain and lost. The divergence time between species was obtained from Time tree database. A family-wide p value (< 0.05) of all the gene families was used to identify those that had a significantly greater rate of evolution. The expanded and contracted gene families were functionally evaluated using combine graph module and KEGG pathway analysis (Kanehisa and Goto 2000) in OmicsBox 3.0.30.

### Selection pressure analysis and phylogeny of WRKY TF family

WRKY genes in each species were identified based on the annotation descriptions and with the presence of WRKY domains in each genome. WRKY proteins were aligned using multiple alignment with fast Fourier transform (MAFFT) alignment in Geneious prime V2021.2, Biomatters Ltd with default parameters. Maximum Likelihood tree of WRKY domain sequences were generated by RAxML (Randomized Axelerated Maximum Likelihood) method, using 1000 bootstrap replicates and generalized time reversible (GTR) GAMMA nucleotide substitutional model (Silvestro and Michalak 2012). MCScanX was applied to identify duplicated WRKY genes. Ka/Ks ratio of each WRKY paralogous gene pair was calculated using simple Ka/Ks calculator module in TBtools (Chen et al. 2020).

## Results

### Genome assembly and annotation

The genomes of all the taxa were assembled using 55–75.9 Gb of PacBio HiFi reads with median read quality ranging from Q31 to Q36 and with an estimated genome coverage depth of 162X to 223X from two PacBio SMRT cells. Scaffolding was performed using 85.1–116 Gb of Hi-C data (250X to 341X coverage of the genome) (Table S2). The previously assembled *C. australis* genome was used as the reference to assign the contigs/scaffolds into pseudochromosomes in two occasions [Chr2 of *C. inodora* collapsed assembly (Table S3) and Chr4 of *C. australasica* (Nakandala et al. 2024)]. The numbering of pseudochromosomes and their orientations were based on the *C. australis* genome (Nakandala et al. 2023b) (Table S3). The sizes of the collapsed nuclear genomes ranged between 315 Mb (*C*. sp) and 391 Mb (*C. inodora*). The size difference between any two haplotypes ranged between 2.1 Mb (*C. australasica*) and 34.5 Mb (*C. inodora*). The N50 of the collapsed nuclear genomes ranged from 29.5 to 35 Mb. The gene completeness (BUSCO) of the assembled genomes was high for all the taxa (Table 1). Information about assembly and annotated gene models of nine pseudochromosomes is given in Table S4.

**Table 1:** The size, contiguity (N50), and gene completeness (BUSCO) of the collapsed and haplotype genome assemblies, including the nine pseudochromosomes and unplaced scaffolds

Species	Assembly	Assembly size (Mb)	Assembly N50 (Mb)	Assembly BUSCO (%)	Non anchored sequence into chromosomes	
					(Mb)	%
*C. australasica*	Collapsed	344.2	35.0	99.1	17.9	5.2
	hap1	321.1	32.3	98.9	40.1	11.7
	hap2	323.2	34.4	99.0	34.1	9.9
*C. australis*	Collapsed	328.5	35.1	98.8	17	5.2
	hap1	325.8	31.4	98.8	27.6	8.5
	hap2	300.0	30.7	97.4	9.6	3.2
*C. garrawayi*	Collapsed	316.8	29.5	98.4	30.8	9.7
	hap1	286.8	30.0	98.3	3.8	1.3
	hap2	312.7	28.9	98.6	30.7	9.8
C. glauca	Collapsed	340.2	33.5	99.3	35.2	10.3
	hap1	318.0	33.3	99.5	17	5.3
	hap2	311.6	32.4	98.4	22.6	7.3
*C. inodora*	Collapsed	391.1	31.5	99.0	83.1	21.2
	hap1	290.8	29.3	98.8	5.8	2
	hap2	325.3	30.3	98.8	37.3	11.5
*C.* sp*	Collapsed	315.0	31.9	98.4	15	4.8
	hap1	298.3	31.5	98.6	11.3	3.8
	hap2	305.0	29.7	98.8	16	5.2

*A distinct form of *C. garrawayi*

**Fig. 2:**
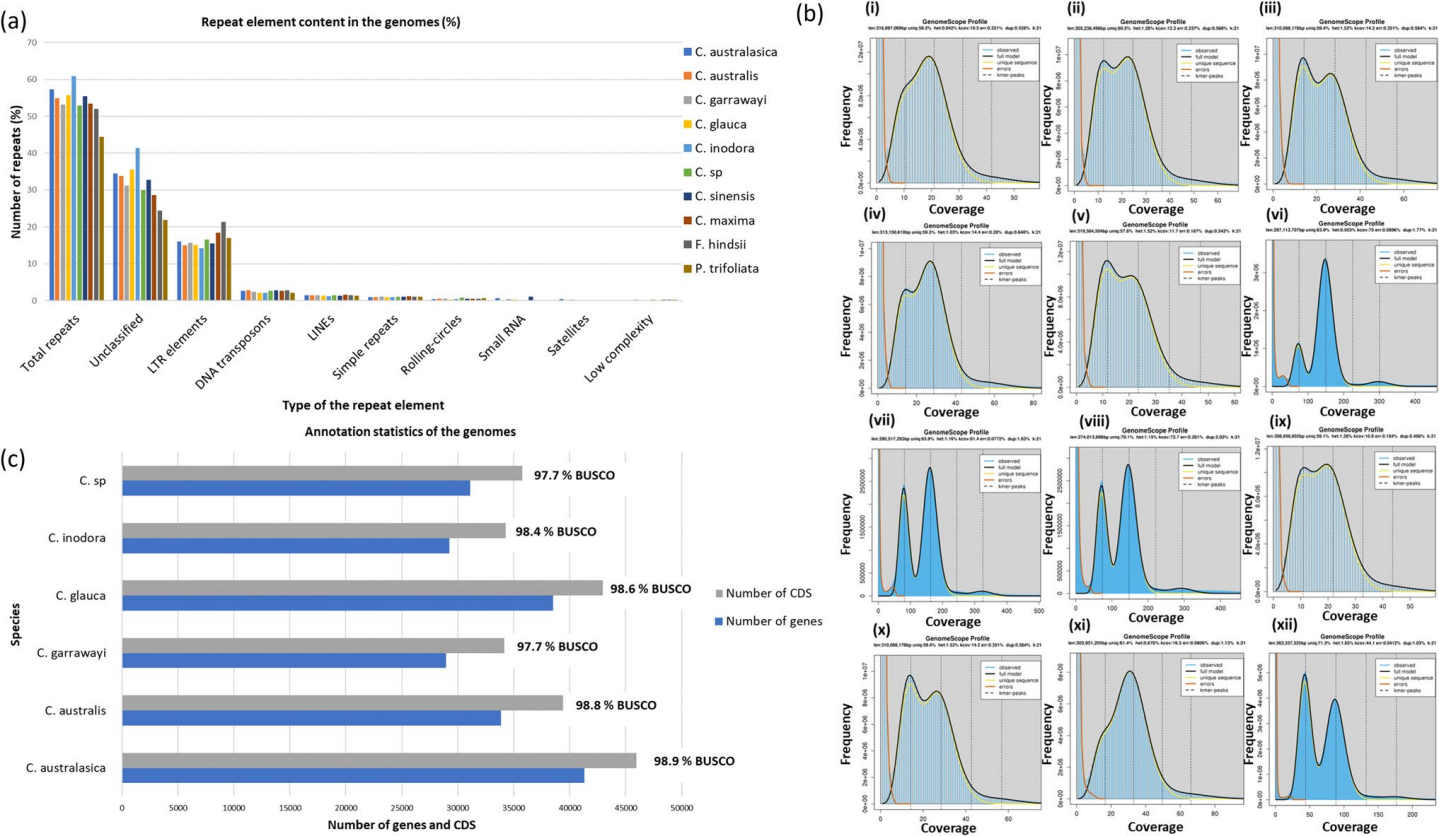
Annotation statistics and heterozygosity of *Citrus* species. (a) Repeat elements were compared among Australian and four other non-Australian, publicly available *Citrus* genomes. More than half of the genome was composed of repeat elements in all the citrus species except in *P. trifoliata*, which had 44.5% repeat content. All the genomes contained a large percentage of unclassified repeat elements followed by LTR elements. The largest unclassified repeat content was found in *C. inodora*, whilst the lowest was found in *P. trifoliata*. The LTR element content was comparable among all the species, whereas *F. hindsii* had a slightly higher LTR repeat content. (b) The heterozygosity of the genomes based on 21 k-mer. (i) *C. australasica* cv 1 – 0.94%, (ii) *C. australasica* cv 2 (Rainbow) - 1.28%, (iii) *C. australasica* cv 3 (Red Champagne) − 1.52%, (iv) *C. australasica* cv 4 (Red Finger lime) - 1.03%, (v) *C. australasica* cv 5 (Ricks red) - 1.52%, (vi) *C. australis* - 0.50% (vii) *C. garrawayi* - 1.16% (viii) *C. glauca* - 1.15% (ix) *C. inodora* - 1.28% (x) *C. ichangensis* - 1.41% (xi) *P. trifoliate* - 0.676% (xii) *A. buxifolia* - 1.65%. The highest heterozygosity was observed for *A. buxifolia*, which is a wild citrus species (1.65%), followed by two *C. australasica* cultivars (red champagne, ricks red) (1.52%). *C. australis* and the citrus related genus, *P. trifoliata* showed low heterozygosity compared to all other species. (c) Genes and CDS predicted in six Australian collapsed nuclear genomes (nine pseudochromosomes and unplaced scaffolds). Genomes had 28,946 genes (34,141 CDS) to 41,304 genes (45,935 CDS). All the genomes had more than 97% annotation completeness

The genomes were structurally annotated to predict the repeat elements and protein coding genes, and their locations in the genomes. All the genomes contained a high number of repeat elements (53% to 60%), where many of them were interspersed repeats, predominantly containing unclassified repeats and LTR elements (Fig. 2a, Table S5). Pearson correlation test was performed to test the correlation between the genome size and the repeat content of the Australian wild limes. The repeat content was found to have a significant, positive correlation with the genome size [Pearson correlation coefficient (r) = 0.985, p < 0.05] (Fig. S1b, S1c). Among the Australian *Citrus*, the *C. inodora* genome had the highest repeat content (60.8%), while *C.* sp. had the lowest repeat content (52.9%). The high repeat content of *C. inodora* was due to the large content of unclassified repeats compared to all other species. We compared the repeat composition of these six Australian *Citrus* taxa with those of four diverse non-Australian species [*C. sinensis* (L.) Osbeck, *C. maxima* (Burm.) Merr, *Fortunella hindsii* (*C. hindsii*)] and *Poncirus trifoliata* a (L.) Raf (*C. trifoliata*), for which high-quality genomes were available. The total repeat content of the Australian species was similar to *C. sinensis*, *C. maxima* and *F. hindsii*. However, the total repeat content of the *P. trifoliata* genome was lower than the other *Citrus* species (44.5%) and that was mainly due to the low percentage of unclassified repeats in the genome (21.9%). The unclassified repeat content of the *F. hindsii* genome was also relatively low (24.5%) similar to that of *P. trifoliata*, however, the LTR elements of *F. hindsii* was the highest among all (Fig. 2a).

Heterozygosity based on a 21 k-mer varied among Australian species (Fig. 2b) and was compared to available data for three other non-Australian wild species including *Atalantia buxifolia*, *C. ichangensis* and *P. trifoliata*. Five *C. australasica* cultivars showed a great variation ranging from 0.94% (*C. australasica* cv 1) to 1.52% (*C. australasica* cv 3 - Red Champagne and *C. australasica* cv 5 - Ricks Red). These results are consistent with our previous study on *C. australasica*, which revealed a high variation in heterozygosity among the five cultivars based on genome wide heterozygous single nucleotide variants (SNVs) (Nakandala et al. 2024). *C. australis* had the lowest heterozygosity (0.50%) among all the wild limes. The k-mer analysis of three other non-Australian wild species revealed a high heterozygosity for *Atalantia buxifolia* (1.65%), which was higher than that of the other wild limes. *P. trifoliata* had a low heterozygosity which was similar to *C. australis,* while *C. ichangensis* had a relatively high heterozygosity (1.41%) (Fig. 2b). The predicted gene models of all the Australian collapsed nuclear genomes were from 28,946 (with 34,141 transcripts in *C. garrawayi*) to 41,304 (with 45,935 transcripts in *C. australasica*) (Fig. 2c, Table S6). The completeness of the annotated gene sets (BUSCO) was high for all the assemblies (97.4 to 99.2%) (Table S6). The difference in number of annotated genes in pseudochromosomes between haplotypes was ranged from a minimum of 70 in *C. australis* to a maximum of 4,089 in *C. australasica*. The CDS sequences of the collapsed and haplotype assemblies of all the species were subjected to functional annotation. Many of the sequences had BLAST hits explaining their protein descriptions. The sequences with no BLAST hits had high coding potentials based on both *Citrus* and *Arabidopsis* coding models (Table S6).

### The inference of gene duplications in genomes and gene collinearity between and within species

**Fig. 3:**
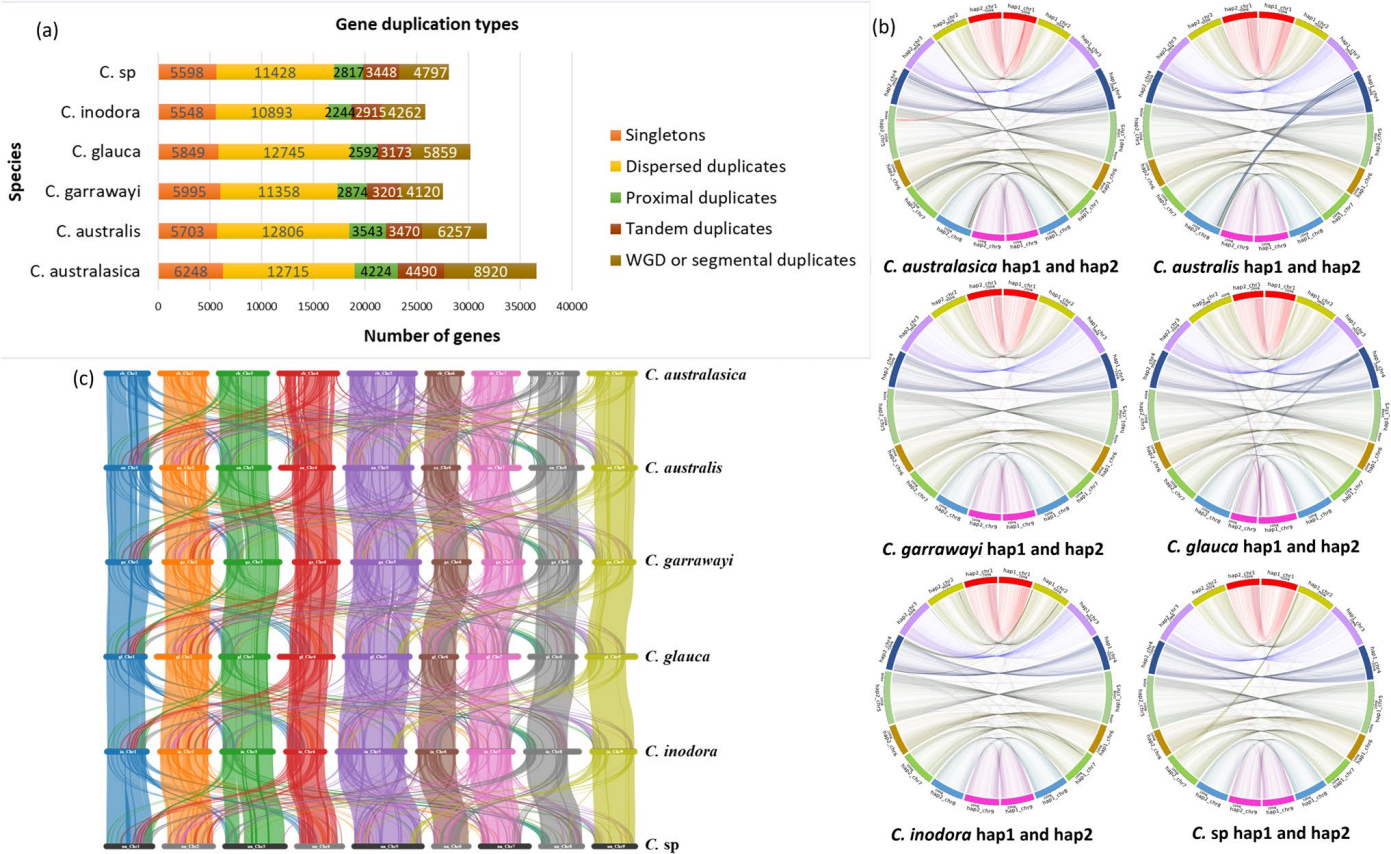
Genes derived from duplication events in genomes, and collinearity within and among different species. (a) Genes duplicated by four different mechanisms. Genes translocated by transposons, that were not adjacent on chromosomes and do not show synteny (dispersed duplicates) were the highest in all genomes. The WGD or segmental duplication was the next dominant mode of duplication in the genomes. Tandem duplicates and Proximal duplicates were nearly similar in all the genomes. (b) Collinear genes between two haplotypes of the same species. (c) Collinear genes between genomes of different species

The genes in the genomes were classified into five categories, based on their copy number, and distributions in the genome by MCScanX algorithm (Fig. 3a). The duplicated genes were predominantly dispersed, and were not adjacent on chromosomes, and did not show conserved synteny. All the genomes had more than 10,000 dispersed duplicates (> 34%), which might have been translocated by transposons in the genomes. The next dominant type of duplicates was segmental / WGD duplicates. The collinear genes derived from WGD events were anchored into collinear blocks by collinearity analysis (Wang et al. 2012). The largest number of segmental / WGD duplicates were identified in the *C. australasica* genome (8,920 – 24%), followed by *C. australis* (6,257 – 19.7%), whilst the lowest number of WGD duplicates were found in *C. garrawayi* (4,120 – 15%). The number of WGD duplicates were comparable, and lower in the other genomes. The other two types of duplicates (proximal and tandem) were also defined as paralogs and were nearly similar in all the genomes. The tandem duplicates were adjacent to each other on chromosomes and the proximal duplicates were near to each other, however separated by a few other genes. All the other genes which were present as one copy (5,548 in *C. inodora* to 6,248 in *C. australasica*) were defined as singletons (Fig. 3a).

The circos plots indicated high collinearity between homologous chromosomes in all the species (Fig. 3b). More than 70% of the genes were found to be collinear between the two haplotypes in all the species (Table S7). The two haplotypes also had structural rearrangements, which were mainly translocations / duplications between them as depicted in Fig. 3b. In *C. australasica*, some collinear blocks were found between Chr7 of hap1 and Chr8 of hap2. Collinear blocks were also found between Chr1 of hap1 and Chr5, Chr8 of hap2. Some collinear blocks were present between Chr7 of hap1 and Chr8 of hap2. Similarly, in *C. australis*, a major translocation was found between Chr4 of hap1 and Chr8 of hap2. In *C. garrawayi*, blocks of genes and their orders in Chr1 were found to be preserved in Chr3, Chr4 and Chr8 of hap2. In *C. glauca*, noticeable translocations were found between Chr4 of hap1 and Chr2, 7, 8 of hap2. In addition, blocks of genes in Chr7, 8, and 9 of hap1, were found to be collinear with Chr2 of hap2. In *C. inodora*, some collinear blocks between Chr2 and 7 of hap1 and Chr4 of hap2 were dominant. In *C*. sp, blocks of genes in Chr1 of hap1 were found to be in a collinear arrangement with Chr3, 4 and 8 of hap2. Additionally, some blocks were also found between Chr2 of hap1 and Chr7 of hap2, and Chr9 of hap1 and Chr5 of hap2, indicating structural rearrangements between the two haplotypes.

Collinear genes are important in deciphering the evolutionary relationships of genomes. The collinear genes between any of the two genomes revealed the genes that were conserved in the same order as their ancestral genomes. The results revealed the presence of the same orthologous genes with conserved orders in the six Australian *Citrus* genomes identified by sequence similarity as described in the methodology (Fig. 3c). Many genes and their orders were conserved between the same corresponding chromosomes of any two species (Figs. 3c, S2). Interestingly, some collinear genes were also found between different chromosomes revealing the structural rearrangements. A conserved pattern of chromosomal locations of the collinear genes could be observed for all the species (Fig. S2). The highest number of collinear genes were shared between *C. australasica* and C. *glauca* (40,320). A high number of collinear genes were also shared by *C. australis* and *C. glauca* (39,787), *C. australis* and *C. australasica* (39,243) and *C. garrawayi* and *C.* sp (39,301) (Table S8). Chr4 showed collinearity with Chr1, 2 between any two species. Apart from the same chromosome, a noticeable number of collinear blocks were found between Chr8 of *C. australis* and Chr2, 3, 4, 6, 7, 9 of *C. glauca* (Fig. S2). Collinear genes were also found between Chr4 and Chr1, 2, 8, 9 in *C. australis* and *C. australasica*, *C. australis* and *C. glauca*, *C. australasica* and *C. glauca,* which were not found between any other two species (Fig. S2). The lowest number of collinear genes were found between *C. inodora* and *C. glauca* (35,755) (Table S8).

**Fig. 4:**
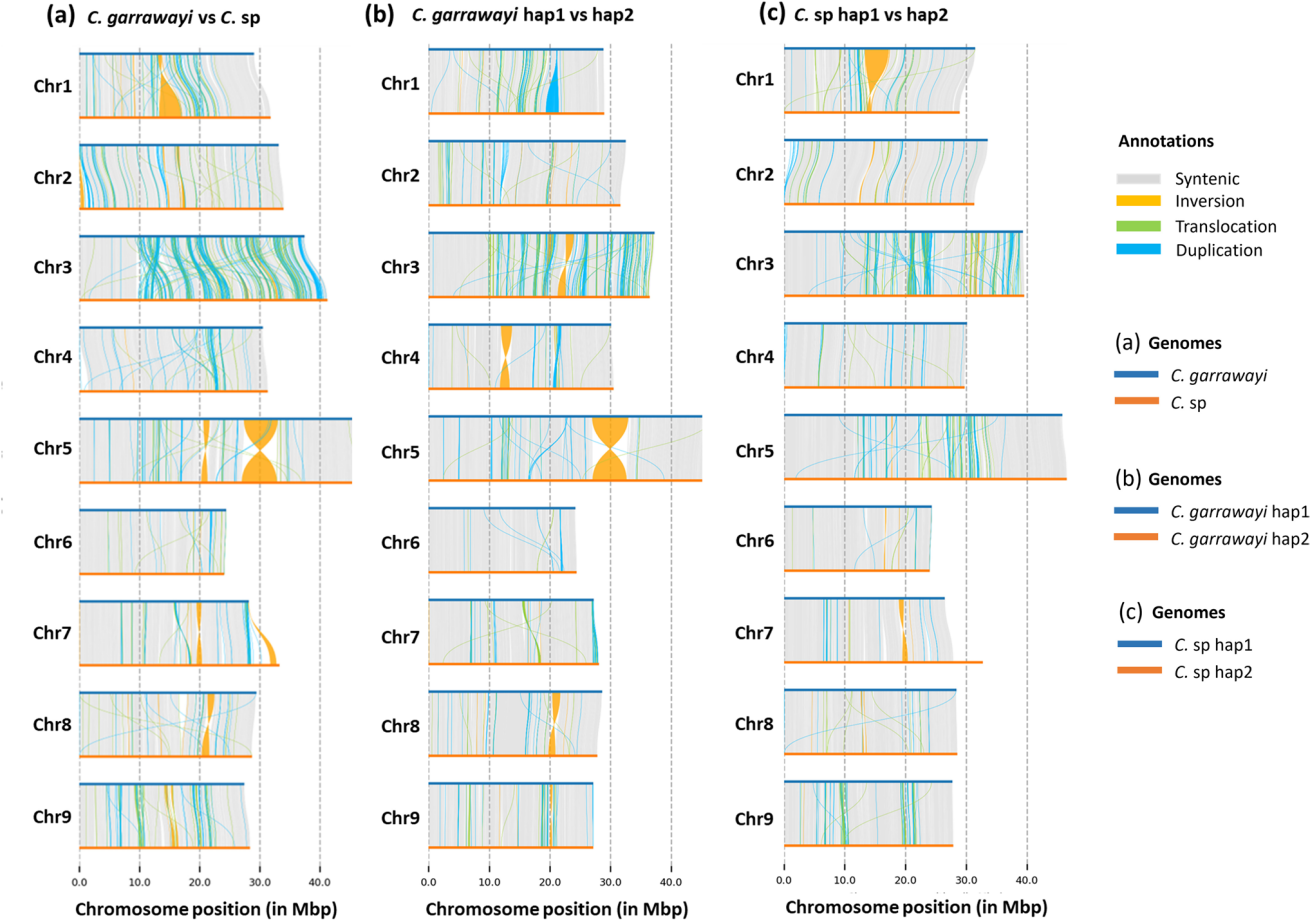
The structural comparison between *C. garrawayi* and *C*. sp genome assemblies across the nine chromosomes. Syntenic regions are shown in grey colour and structural variations such as inversions, translocations, and duplications are shown in orange, green, and blue colour respectively. (a) The comparison between *C. garrawayi* and *C*. sp collapsed genomes. (b) The comparison between *C. garrawayi* haplotypes. c The comparison between *C*. sp haplotypes. Chr3 had many translocations and duplications between the collapsed genomes and between the haplotypes. Chr6 had more syntenic regions. A large-scale inversion was found between the two collapsed genomes and between *C. garrawayi* haplotypes in Chr5

The undetermined *C*. sp was previously identified as a closely related, but distinct form of *C. garrawayi*, using 86 single copy nuclear genes (Nakandala et al. 2023a). Comparison of this distinct accession with the conventional form, using complete whole genomes now revealed large structural variations between the two forms of this species (Fig. 4a). A large-scale inversion was identified in Chr5, and small-scale inversions were found in all chromosomes. Chr3 had the largest number of structural variations represented by translocations and duplications, while Chr6 had more syntenic regions and less structural variations. In total, 10,619 syntenic regions, 123 inversions and 3,980 translocations were found between the two genomes. Local sequence variations such as insertions (973), deletions (995), 898 copy gains, 820 copy losses and 3,023 highly diverged regions were found across the two genomes.

The haplotype comparison of *C. garrawayi* (Fig. 4b) and *C*. sp (Fig. 4c) revealed many structural variations within *C. garrawayi* compared to *C*. sp. A total of 81 inversions, 635 insertions, 650 deletions, 589 copy gains and 624 copy losses were found between *C. garrawayi* haplotypes, whereas it was 70 (inversions), 589 (insertions), 578 (deletions), 522 (copy gains), and 479 (copy losses) between *C*. sp haplotypes. The number of translocations were high between *C*. sp (2403), compared to *C. garrawayi* (2164). Moreover, the number of duplications were also higher in *C*. sp than in *C. garrawayi*. Similar to the collapsed genomes, the Chr3 of both *C. garrawayi* and *C*. sp haplotype assemblies had the largest number of total structural variations, while Chr6 had the lowest number of variations.

### Evolutionary events and comparative analysis of gene families

**Fig. 5:**
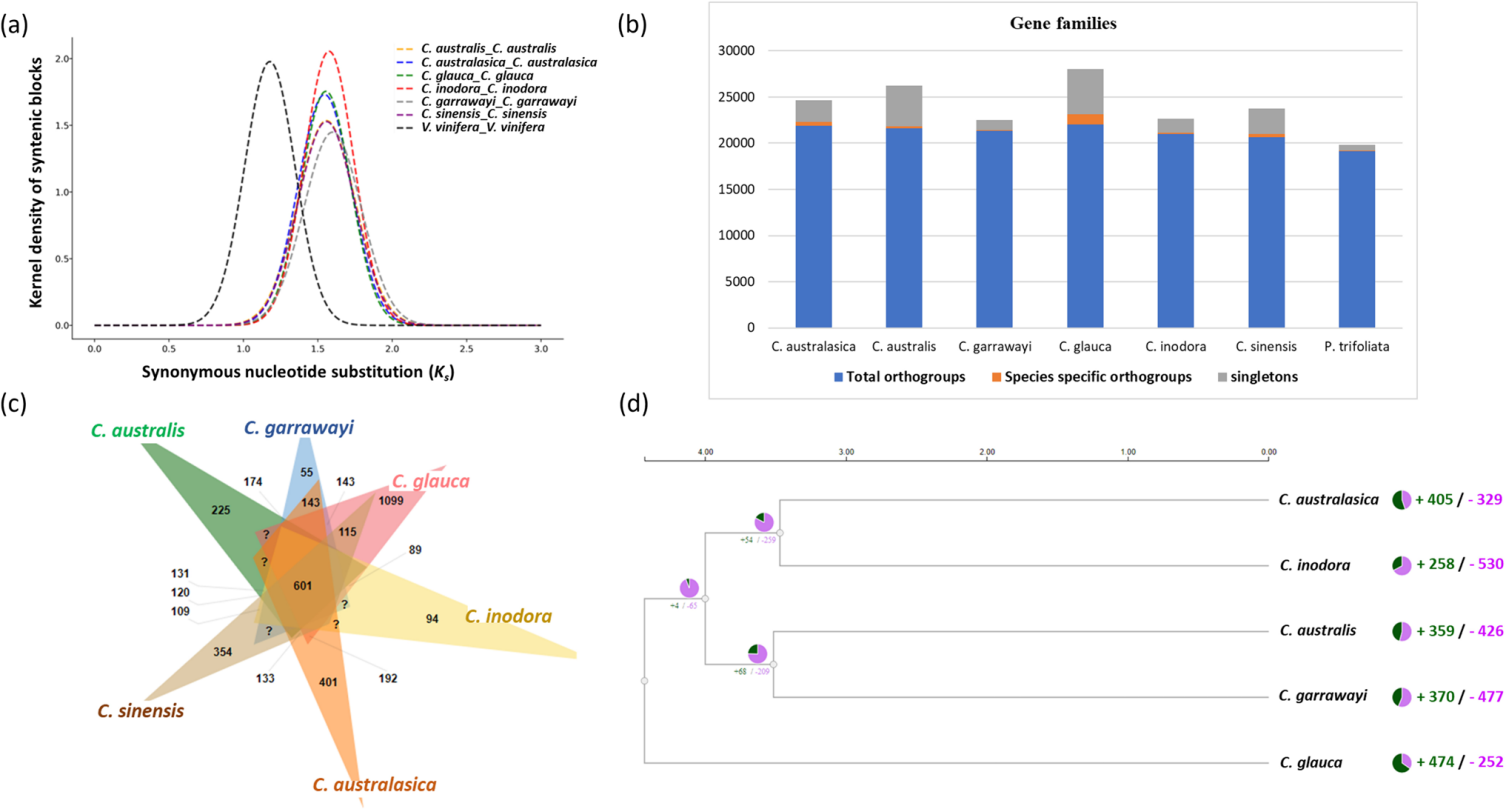
Evolutionary events and comparative analysis of gene families among *Citrus* species. (a) Ks distribution of paralogous gene pairs of *C. australasica*, *C. australis*, *C. garrawayi*, *C. glauca*, *C. inodora*, *C. sinensis* and *V. vinifera*. The ks peaks were calculated using WGDI tool. (b) Comparison of gene families and singletons among seven citrus species. The total number of orthogroups/gene families (conserved and unique) are indicated in blue. Species specific unique orthogroups/gene families are depicted in orange. Singletons in each species are indicated in grey colour. (c) Venn diagram showing unique and shared orthogroups/gene families among seven citrus species including *C. sinensis* and *P. trifoliata*, using Orthofinder. The diagram with *P. trifoliata* is shown in Fig. S5. (d) Gene families underwent expansions and contractions in Australian native citrus species identified using CAFE5 (Mendes et al. 2020). The pie charts show the number of gene families expanded (green) and contracted (pink) in each species. The Venn diagram for gene families, phylogenetic tree and pie charts of expansions and contractions were generated using different modules in Orthovenn3 tool (Sun et al. 2023)

To investigate the polyploidization events in the evolution of Australian *Citrus* species, the synonymous substitutions (Ks) were estimated for each species. *C. sinensis* and *V. vinifera* were used as references. The peaks for the distributions of Ks for paralogous gene pairs of each *Citrus* species were clearly seen at Ks =  ~ 1.5 (*C. australasica* = 1.55, *C. australis* = 1.56, *C. garrawayi* = 1.59, *C. glauca* = 1.55, *C. inodora* = 1.58, *C. sinensis* = 1.56) (Figs. 5a, S3). The results are consistent with previous studies for *Citrus* [*C. clementina* Ks peak = 1.5; (Wu et al. 2014)] and *Citrus* related genomes [*P. trifoliata* Ks peak = 1.5; (Peng et al. 2020)]. The reference *Vitis vinifera* had the Ks peak at 1.18. The reference *V. vinifera* has undergone only one polyploidization event (whole-genome triplication) in its history (the γ-WGT event) (Almeida-Silva and Peer 2023). Based on our results (a single Ks peak in Australian citrus), there is not any evidence for subsequent WGD events in Australian citrus in addition to the γ-WGT event, which was shared by all eudicots in their evolutionary history. Since the species evolutionary rates are considerably different (Sun et al. 2022), it is possible that the different Ks peaks of *V. vinifera* and the Australian citrus might not represent different evolutionary events. To determine the divergence between Australian *Citrus* and Asian *Citrus,* the Ks were estimated between the orthologs of *C. australis* and *C. sinensis,* and a peak at Ks = 0.032 (Fig. S4) was observed for this divergence event. With the assumption of a neutral substitution rate r to be 1–2 × 10^−9^ in citrus as used by Wu (Wu et al. 2014), the time that this divergence happened was estimated to be 8–16 MYA.

### Conserved and unique gene families

To explore the unique gene families, the data was collected from the five main Australian native citrus taxa from this study, another representative citrus species (*C. sinensis*), and the related genus *P. trifoliata*, for which high quality genomes and chromosome-level annotations were available. 92.9% of the total genes were assigned to 27,262 orthogroups (Fig. 5b). In total, 15,536 gene families, containing 127,511 genes were shared among the seven species, which represents their core proteome. Of these species, *C. glauca* had the highest number of species-specific gene families (orthogroups) (1,099), encompassing 5,147 genes, and 4,579 singletons which were not assigned to orthogroups (Fig. 5b, c). The second highset number of unique gene families was identified in *C. australasica* with 401 gene families including 8,233 genes and 2,184 singletons. *C. garrawayi*, *P. trifoliata* and *C. inodora* had the lowest number of species-specific gene families in them (*C. garrawayi*—55, *P. trifoliata*—93, *C. inodora*—94) (Fig. S5).

All the species had unique genes enriched in two main pathways related to purine and thiamine metabolism (Table S9). Each species had less than 32 unique genes associated with these two pathways except for *C. glauca* which had 117 unique genes for purine metabolism and 96 unique genes for thiamine metabolism which is exceptionally high compared to the other species (Table S9). In the purine metabolism pathway, allantoinase (ALN) is a key enzyme which converts allantoin to allantoate (Kaur et al. 2023) (Fig. S6). ALN is one of the unique genes identified in *C. glauca*, which is structurally different from the corresponding genes in the other native species. In *C. glauca*, there were two CDS encoding ALN. One CDS is similar to the ALN CDS found in the other species, while the other CDS is structurally different and unique. The unique ALN CDS in *C. glauca* is longer than the others (3,447 bp), containing only four exons, three introns and 72.38% GC content. The common ALN CDS of other species and *C. glauca* ranged from 1,488 to 1,524 bp, containing 15–17 exons, 14–16 introns and 44.16–44.42% GC contents. The corresponding unique protein of *C. glauca* had 1,148 amino acids (aa), whereas the common ALN protein of all the species had 495 to 507 aa (*C. inodora*—507 aa, *C. garrawayi*—506 aa, *C. australis*—495 aa, *C. australasica*—507 aa). Other than purine and thiamine metabolism, *C. glauca* specific genes were enriched in biosynthesis of cofactors, pyruvate metabolism, glycine, serine and threonine metabolism, butanoate metabolism, glyoxylate and dicarboxylate metabolism and cysteine and methionine metabolism (Table S9). For *C. glauca* specific genes, the enriched GO terms included the molecular functions corresponding to transferase activity, hydrolase activity and oxidoreductase activity. The proteins encoded by unique genes in *C. glauca* were mostly present in the plasma membrane (Table S10).

In addition to purine and thiamine metabolism pathways, gene families unique to *C. australasica* were predominantly enriched in terpenoid backbone biosynthesis, monoterpenoid biosynthesis, starch and sucrose metabolism, glutathione metabolism, and the Toll-like receptor (TLR) signalling pathway (Table S9). The top enriched molecular functions included protein binding, and hydrolase activity, and many gene products were identified to be present in cell nucleus (Table S10). The top enriched pathways of unique genes in *C. australis* were associated with biosynthesis of cofactors, glycerolipid metabolism, tryptophan metabolism, phenylpropanoid biosynthesis, plant-pathogen interaction, starch, and sucrose metabolism (Table S9). The molecular functions were mainly related to hydrolase activity, protein binding and nucleic acid binding (Table S10). *C. garrawayi* had unique genes enriched in plant-pathogen interactions, phenylpropanoid biosynthesis, starch and sucrose metabolism, terpenoid backbone biosynthesis and monoterpenoid biosynthesis, while those of *C. inodora* corresponded to tryptophan metabolism and metabolism of xenobiotics by cytochromosome P450 in KEGG pathways (Table S9).

### Gene family expansion and contraction

The likelihood approach for gene family evolution rates revealed the gene families which underwent significant expansion or contraction (p < 0.05) in a specific lineage when compared to the most recent common ancestor. The number of expanded gene families in *C. australasica* (405) and *C. glauca* (474) were higher than their contracted gene families, however, the reverse was true for *C. inodora*, *C. australis* and *C. garrawayi* (Fig. 5d). In total, 405 expanded gene families were identified in *C. australasica* with notable enrichment in pathways of plant-pathogen interaction, thiamine metabolism and purine metabolism (Table S11) and in the GO terms of transferase activity and hydrolase activity (Table S12). For C. *australis*, the expanded gene families were mostly enriched in plant-pathogen interaction, steroid hormone biosynthesis and tryptophan metabolism in KEGG pathways (Table S11) and protein binding, hydrolase activity in the GO terms (Table S12). In *C. garrawayi*, the top three significant pathways associated with expanded genes included phenylpropanoid biosynthesis, purine metabolism and thiamine metabolism (Table S11) and were enriched in protein binding and hydrolase activity in GO terms (Table S12). In *C. glauca*, the expanded gene families were mostly enriched in tryptophan, purine, and thiamine metabolism in KEGG pathways (Table S11) and nucleic acid binding and protein binding in GO terms (Table S12). For *C. inodora*, the topmost pathways associated with expanded gene families included steroid hormone biosynthesis, purine, tryptophan metabolism (Table S11) and GO terms included nucleic acid binding and transferase activity (Table S12).

The above results indicated that both *C. australasica* and *C. australis* had gene families with a relatively high number of gene copies for plant-pathogen interactions. Among the expanded gene families related to plant-pathogen interactions, 37 genes in *C. australasica* and 51 genes in *C. australis* were related to Disease Resistance Protein RPS2 (RPS2). *C. garrawayi*, *C. glauca* and *C. inodora* had 25 genes, 15 genes and 15 genes, respectively, related to RPS2 protein. In all the species, most of the RPS2 proteins contained nucleotide-binding site (NBS), and leucine-rich repeats (LRRs) or ARC domain. In addition to RPS2 protein, the other expanded genes in *C. australasica* were related to mitogen-activated protein kinase 1, 3-Ketoacyl-CoA Synthase (KCS) and Glycerol Kinase (GK). In *C. australis*, the other expanded genes were associated with mitogen-activated protein kinase 1, GK, LRR Receptor-Like Serine/Threonine-Protein Kinase (FLS2), and Senescence-Induced Receptor-Like Serine/Threonine-Protein Kinase (FRK1). In *C. garrawayi*, the other expanded genes were associated with KCS, FRK1 and Cyclic Nucleotide Gated Channel, Plant (CNGC). The expanded genes in *C. glauca* were associated with KCS, GK, FRK1, CNGC, Pathogenesis-Related Protein 1 (PR1) and Elongation Factor Tu (Tuf, TUFM).

The contracted gene families of *C. australasica* were mainly enriched in biological processes related to GO terms of signal transduction, protein phosphorylation and response to stress, and molecular functions related to GO terms of ATP binding, and protein binding (Table S13). For *C. australis*, the GO terms included regulation of cellular process, cellular nitrogen compound biosynthetic process, protein modification process (biological processes) and transferase activity, protein binding (molecular functions). In *C. garrawayi*, most of the contracted genes were involved in biological processes including RNA metabolic process, regulation of cellular process, DNA integration and molecular functions such as protein binding and oxidoreductase activity. Those of *C. glauca* were mainly involved in protein phosphorylation and RNA, DNA metabolic processes and molecular functions such as nucleic acid binding and hydrolase activity. *C. inodora* had contracted gene families enriched in protein phosphorylation, multicellular organismal process and anatomical structural development as biological processes, and protein binding, hydrolase activity as molecular functions (Table S13).

### Gene families related to immunity and abiotic stress tolerance

**Fig. 6:**
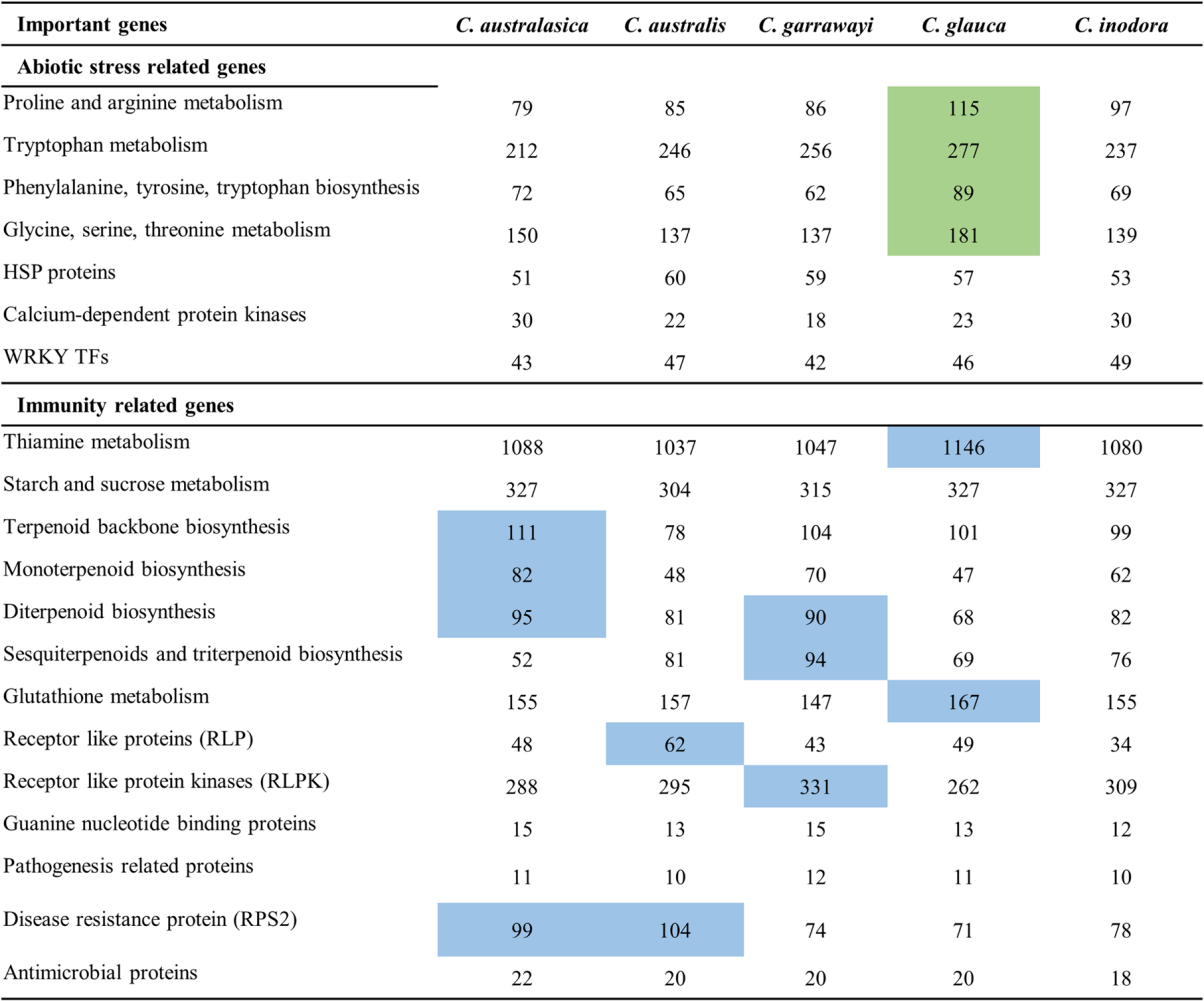
Total number of genes related to abiotic stress and immunity related genes among Australian native citrus species. Species with proportionately high gene numbers for abiotic stress related proteins or pathways are highlighted in green colour. Species with proportionately high gene numbers for immunity related genes are highlighted in blue colour

The total genes related to some important immunity related gene families / pathways and abiotic stress related genes / pathways were compared among the five main native taxa (Fig. 6). *C. glauca* had a high number of genes related to the abiotic stress related KEGG pathways of proline and arginine metabolism, tryptophan metabolism, phenylalanine, tyrosine and tryptophan biosynthesis, and glycine, serine, threonine metabolism. The number of genes for HSP proteins, calcium-dependent protein kinases and WRKY TFs were comparable among the species. Previous studies have reported that a cold-regulated gene LOW-TEMPERATURE-INDUCED 65 (LTI65), which is induced by C-repeat/DREB binding factor (CBF) genes, are positively selected in two mostly cold-tolerant species *P. trifoliata* and *C. ichangensis* (Peng et al. 2020). We identified the homologs of *P. trifoliata* LTI65, in Australian citrus and *C. sinensis* using Orthofinder. We found the orthogroup for this gene in *P. trifoliata* (OG0009805) and found the corresponding orthologues in other species. This revealed one ortholog in each species and the gene tree for this orthogroup revealed that these orthologues arose in two separate events (Fig. S7). *C. glauca,* which can survive under extreme cold temperatures was identified with a homolog of LTI65, which is g28904.t1, and this gene would be a good candidate for future research on its expression and evolutionary selection in response to cold tolerance. When considering immunity related genes, *C. glauca* had the highest number of genes for thiamine metabolism and glutathione metabolism pathways (Fig. 6). *C. australasica* had a relatively large number of genes for terpenoid backbone biosynthesis, monoterpenoids and diterpenoid biosynthesis pathways and disease resistant protein (RPS2). *C. australis* had a large number of genes encoding receptor like proteins (RLP) and, and *C. garrawayi* had a high number of genes related to diterpenoids, sesquiterpenoids and triterpenoid biosynthesis pathways and receptor like protein kinases (RLPK).

### WRKY TF family

WRKY TFs are known to have pivotal roles, modulating abiotic and biotic stress tolerance in plants. The number of genes for WRKY TFs were comparable among the five main Australian native citrus species included in our study (Fig. S8). The WRKY family was represented by 42 genes in *C australasica*, 48 genes in *C. glauca*, 48 genes in *C. australis*, 50 genes in *C. inodora,* and 42 genes in *C. garrawayi*. The WRKY genes were categorized into three main groups (GI, GII and GIII) based on the number of WRKY domains and the type of zinc-finger domains. Group I had two WRKY domains and C_2_H_2_-type zinc-finger domain. Group II had one WRKY domain and C_2_H_2_-type zinc-finger domain. Group III had one WRKY domain and C_2_HC-type zinc-finger domain. Group II was further categorized into five sub-groups, based on the type of zinc-finger structure and their phylogenetic position. Group IIa consisted of C-X_6_-C-X_23_-H-X-H zinc-finger structure, with X being an arbitrary amino acid). Group IIb consisted of C-X_5_-C-X_23_-H-X-H. Group IIc contained C-X_4_-C-X_23_-H-X-H. Group IId contained C-X_5_-C-X_23_-H-X-H and Group IIe consisted of C-X_4_-C-X_23_-H-X-H (Fig. S8). All the genes had the conserved WRKYGQK domain except for ten genes within Group IIe, which had WRKYGKK domain.

The duplication events of WRKY genes were analysed to reveal the expansions of WRKY TF family in each species. Among the WRKY TF genes, 14 gene pairs in *C. glauca* (58.3%), 11 gene pairs in *C. australis* (45%), *C. inodora* (44%) and *C. garrawayi* (52.3%) and 10 gene pairs in *C. australasica* (47.6%) were involved in duplication events (Table S14). All the duplicated genes were derived from WGD / segmental duplication events. To further understand the adaptive evolution of WRKY genes, the Ka/Ks (non-synonymous / synonymous) ratios of all gene pairs were calculated. All of them had Ka/Ks < 1, indicating that the purifying selection had acted on the evolution of WRKY genes and some duplicated gene pairs had no Ka/Ks values, which might be due to the low sequence divergence (Table S14).

## Discussion

The lack of high-quality haplotype-resolved genomes for Australian wild limes has hindered the discovery of important, novel gene repertoire in Australian *Citrus*. Here we provide the first report in which the haplotype-resolved, chromosome-level genomes of six Australian wild *Citrus* taxa were compared. All species yielded highly contiguous collapsed and haplotype-resolved assemblies, with high assembly and annotation completeness, indicating that both assembly and annotation approaches have succeeded in capturing most of the genes that were previously identified in the viridiplantae lineage. Some pseudo-chromosomes in all the species could not be assembled completely with telomeres at both ends due to the presence of large tandem arrays of satellite repeats and extensive rRNA repeats at terminal regions of chromosomes. This indicates that in addition to PacBio HiFi and Hi-C data, other additional types of data such as ultra-long ONT data may be needed to achieve more complete and gapless assemblies for highly repetitive plant genomes such as *Citrus* (Fann et al. 2001; He et al. 2020). The two haplotype assemblies were found to have structural differences in terms of length, copy number of annotated genes, and structural rearrangements such as translocations. The majority of the genes were found to be in a collinear arrangement, where those genes and the order of the genes were conserved between the two haplotypes. Size variations and copy number variations between haplotypes have been extensively reported in other studies (Guk et al. 2022).

The amplification of retrotransposons has been identified as a major reason for genome expansions other than polyploidization (Devos et al. 2002). We observed a strong, positive and statistically significant correlation between the whole genome sizes of the Australian limes (including the unplaced scaffolds) and their total repeat contents. This observation was not made for the other non-Australian citrus species that we considered in this study due to the lack of information about the completeness of those assemblies. The size of the genomes is based on the assignment of the scaffolds into the nuclear genome and that can vary depending upon the assembly approach. Therefore, the correlation between the genome size and their repeat content could be accurately deduced only for the genomes assembled in this study. Among all the genomes considered in this study, *C. inodora* had the highest repetitive content. The unclassified repeat elements dispersed across nine chromosomes and unplaced scaffolds were the most abundant type of repeat elements, presumably due to a recent expansion in novel TE elements, which could not be reliably classified by repeat detection software. In contrast, the *C*. sp had the lowest unclassified repeat content, which was consistent with having the lowest genome size among the Australian Citrus. Transposon expansion has been reported to be responsible for variations in genome sizes in other *Citrus* species and related genomes (Yang et al. 2023).

The heterozygosity of genotypes is an indication of genetic diversity within a population. In general, wild plant populations are known to have high levels of heterozygosity compared to domesticated or cultivated plants, which have enhanced their resilience to biotic and abiotic stresses (Shirasawa et al. 2021). Heterozygosity analysis of wild limes based on k-mer approach in the present study revealed large variation in *C. australasica*, consistent with our previous findings (Nakandala et al. 2024), which supports its high morphological diversity. *A. buxifolia*, *C. ichangensis* and two *C. australasica* cultivars had high levels of heterozygosity indicating their high allelic diversity, which may have resulted from natural crosses. *C. glauca* was expected to have high heterozygosity based on the stressful environments in which it occurs, however, the accession used in this study had relatively low heterozygosity. Other species with low heterozygosity were *C. australis*, *C. inodora*, *C. garrawayi*, three accessions of *C. australasica* and *P. trifoliata.* When considering non-Australian *Citrus*, pure genotypes, such as citrons (*C. medica*), have shown low levels of heterozygosity due to the cleistogamy of their flowers, which favours self-pollination and fertilization. By contrast, genotypes such as sweet orange, grapefruits, some mandarins, sour orange, lemons and limes have shown high levels of heterozygosity, due to their hybrid origins and interspecific gene flow (Wu et al. 2018). However, analysis of more accessions will be required to better understand the genetic diversity within these species, and to explore how it associates with environmental adaptation and evolution of the species.

Gene duplication, which is thought to be a key driving force in evolution, has long been studied in many Angiosperms (Cui et al. 2006). Whole genome duplication / polyploidization events and subsequent karyotype formation have been well-documented in citrus. *Citrus* has evolved from an ancient paleohexaploid ancestor and is believed to have undergone chromosomal fissions and fusions to form nine chromosomes (Feng et al. 2021). Ks based methods have revealed that *Citrus* species such as *C. sinensis*, *C. clementina* and a few related genera including *P. trifoliata* and *A. buxifolia* have undergone only one polyploidization event in history which is common to all eudicots (Peng et al. 2020; Wu et al. 2014; Xu et al. 2013; Yuan et al. 2019). The present study confirmed that no lineage specific WGD events have occurred in Australian *Citrus* after the divergence from Asian *Citrus*, which was estimated to have happened around 8–16 MYA. Similar estimates for divergence of Australian *Citrus* from Asian *Citrus* have previously been reported based on molecular clock dating analysis using chloroplast genomes and fossil calibrations (Carbonell-Caballero et al. 2015; Manafzadeh et al. 2014).

In addition to WGD, other types of mechanisms such as dispersed, transposed, tandem, and proximal duplications can multiply genes in a genome generating paralogous gene pairs (Chen et al. 2023a). The most prevalent type of gene duplication in Australian wild limes is dispersed duplication, where the duplicated genes are not adjacent in the genomes or not found within homeologous chromosome segments. Dispersed duplicates can be generated through relocation of a tandem or segmental duplication (Ganko et al. 2007). WGD / segmental duplicates have further contributed to gene duplications in Australian limes. Most of the other eudicot genomes which have undergone lineage specific WGD events, in addition to the common WGT event in history, have retained more genes created through WGD events. This indicates that the dispersed duplication has had a profound impact on the gene repertoire of Australian *Citrus*, while it was the WGDs that have a major impact for the retained duplicates in other eudicots that went through lineage specific WGDs (Wang et al. 2012).

The collinearity analysis indicated a high synteny in genes and their arrangements between *C. australasica* and C. *glauca, C. australis* and *C. glauca, C. australis* and *C. australasica,* and *C. garrawayi* and *C.* sp. The close relationships among *C. glauca*, *C. australis*, *C. garrawayi* and *C*. sp were also supported by our previous phylogeny based on the sequence similarity of 86 single copy nuclear genes (Nakandala et al. 2023a). The syntenic regions and collinear arrangements present in the nuclear genomes of *C. garrawayi* and *C*. sp., and their close proximity in nuclear phylogeny, indicate that these two *Citrus* are closely related. However, the structural differences such as translocations, inversions, and duplications between the two genomes reveal that these two *Citrus* are distinct from one another and support their morphological diversity. Current results support our previous findings that these are distinctly different accessions of the same species (Nakandala et al. 2023a).

*C. glauca* occurs across a vast geographical area and has several adaptations to thrive in unfavourable environments. This species drops leaves in drought conditions and performs photosynthesis using the leafless twigs. Leaves of this species have a thick cuticle and deep sunken stomata (Scora and Ahmed 1995). In addition, it develops a large underground root system before it develops the aboveground parts of the tree, which is another adaptation to drought (Douglas 2017). *C. glauca* has other important features such as cold and salt tolerance, excess boron tolerance, and nematode resistance. The involvement of secondary metabolites in response to various stresses is different among species (Khan et al. 2017). Comparative gene family analysis revealed the highest number of species-specific gene families in *C. glauca*, which might explain its resilience to biotic and abiotic stresses. Unique gene families were predominantly enriched in purine and thiamine metabolism, and the number of unique genes for these two metabolic pathways is higher than for any other species. Purine and thiamine metabolism were also two significantly expanded gene families in all species revealing their importance in Australian *Citrus*. Previous studies have shown that thiamine can induce resistance to salinity stress, by enhancing calcium signal transduction, and activating WRKY TFs, which modulate cellular and molecular defence mechanisms in plants. Thiamine is known to enhance the production of photosynthetic pigments and nitric oxide, which in turn alleviate oxidative stress in plants in response to boron toxicity. Thiamine is also involved in plant systemic acquired resistance to pathogens via upregulation of pathogen-related genes (El-Shazoly et al. 2019; Kaya et al. 2020; Li et al. 2022). Purine metabolism is involved in enhancing drought and salinity tolerance in plants (Lescano et al. 2016; Watanabe et al. 2014; Zhang et al. 2021). An ALN encoding gene, which catalyses allantoin, was found to have structural differences in *C. glauca* compared to other species. Increased levels of allantoin have been shown to improve drought and salinity tolerance, by inducing pathways including abscisic acid, brassinosteroid biosynthesis and jasmonic acid, and by activating stress responsive genes and reactive oxygen species (ROS) scavenging enzymes in plants. Structural difference of allantoinase might be involved in regulating allantoin expression under stress conditions in *C. glauca*. Further research on expression of this gene is required to better understand its involvement in response to drought and salinity in *C. glauca*.

Genes for amino acid metabolism such as glycine, serine and threonine metabolism, and proline and arginine metabolism, have been found to play important roles in maintaining the structural integrity of cellular proteins, and osmoregulation which helps plants to cope with high temperature stress (Chen et al. 2023b). Moreover, the aromatic amino acids such as phenylalanine, tyrosine, and tryptophan are crucial for protein synthesis and to mitigate biotic and abiotic stresses in plants (Yang et al. 2020). *C. glauca* was identified as having 42 unique genes for glycine, serine and threonine metabolism. Tryptophan metabolism was also identified as an expanded gene family in *C. glauca*. Furthermore, the total number of genes for KEGG pathways including proline and arginine metabolism, aromatic amino acids biosynthesis and metabolism, glycine, serine and threonine metabolism pathway was higher in *C. glauca,* than in any other Australian native species for the corresponding pathways. These findings may help future investigations of the involvement of these genes in response to stress conditions in *C. glauca* through gene expression analysis and metabolomics approaches.

*C. australasica* and *C. australis* show resistance / tolerance to HLB (Alquézar et al. 2021; Weber et al. 2022). Terpene biosynthesis is upregulated in HLB tolerant plants (Wang et al. 2016), and unique genes for the terpenoid backbone and monoterpenoid biosynthesis have now been identified in *C. australasica*. In addition, 17 species-specific genes in *C. australasica* and eight species-specific genes in *C. australis* related to starch and sucrose metabolism might potentially be involved in HLB resistance. Previous studies on HLB have revealed increased callose deposition in phloem elements, reduction in the movement of photo assimilates into sink tissues, and starch accumulation in leaves in HLB infected plants, compared to HLB resistant plants. This results in nutrient deficient symptoms and growth retardation in infected trees. Starch accumulation in source tissues inhibit photosynthesis and leads to leaf chlorosis in infected trees. The up-regulation of genes for callose deposition, starch accumulation and down-regulation of genes for starch breakdown are primarily associated with disease symptoms in HLB susceptible plants. However, the resistant plants do not show any of these symptoms (Boava et al. 2017; Weber et al. 2022). The genes identified in *C. australasica* and *C. australis* related to starch biosynthesis might not be expressed as in susceptible plants, potentially explaining the absence of starch accumulation. Moreover, it is possible that the unique metabolic genes for starch and sucrose metabolism might be involved in efficient transportation of photo assimilates to sink tissues, thereby maintaining plant health and growth.

Glutathione metabolism genes are known to be upregulated in response to psyllid attacks (Wei et al. 2021). Glutathione-S-transferases (GSTs) are antioxidants that can mitigate HLB disease symptoms through controlling the overproduction of ROS (Weber et al. 2022). Moreover, the Toll-like receptors (TLRs) can recognize pathogen associated molecular patterns (PAMPs) and can activate downstream defence responses in plants (Duan et al. 2022). The unique genes related to glutathione metabolism and TLR signalling in *C. australasica* might be potential candidates for future research on their roles in response to HLB in Australian limes. Plant immunity is of two types; PAMP triggered immunity (PTI) and effector triggered immunity (ETI) (Chen et al. 2019). The expanded gene families in *C. australasica* and *C. australis* were primarily enriched in plant-pathogen interactions with the majority of the genes encoded RPS2 protein, leading to a hypersensitive response (HR), which is a type of ETI. HR prevents disease spread by localized cell death at the area of pathogen attack (Balint‐Kurti 2019). RLPs and RLPKs encoding genes are higher in number in *C. australis* and *C. garrawayi,* respectively, which might mediate defence responses against pathogens. WRKY TFs mediate biotic and abiotic stress responses in plants in three ways (Long et al. 2023). They can mitigate stresses by directly binding to W-box cis-acting elements of stress-associated genes and regulate their expression, or by inducing the signalling cascades which mediate the expression of downstream stress-associated genes or interact with proteins to modulate the production of defence-related chemicals. Different WRKY TFs are involved in mitigating various abiotic stresses including drought, salinity, and cold (Long et al. 2023). The analysis of WRKY TFs indicates that WRKY genes in Australian *Citrus* have expanded through WGD / segmental duplication and have been subjected to purifying selection during evolution. More than 50% of the WRKY genes in *C. glauca* might have been generated from duplication events. The WRKY genes in other citrus such as *C. reticulata* and *C. sinensis* and many other plant species have also evolved primarily through purifying selection and then expanded through segmental or tandem duplication events (Maheen et al. 2023; Song et al. 2023; Waqas et al. 2019; Xi et al. 2023; Yu et al. 2022). The characterization of WRKY gene family in Australian *Citrus* helped in understanding different categories of WRKY genes and their evolution in Australian *Citrus*. Future research is needed to identify their involvement in response to stress.

In summary, the availability of high-quality genomes for Australian *Citrus* has laid the foundation to identify and validate important genes and pathways involved in abiotic and biotic stresses. It is imperative to study their expression patterns in response to specific stresses to validate their involvement in plant response, which will further enable the introgression of validated genes into cultivated *Citrus* through conventional breeding or genetic engineering approaches.

## Supplementary Information

Below is the link to the electronic supplementary material.

Supplementary file1 (DOCX 6623 kb)

Supplementary file2 (XLSX 60 kb)

## Data Availability

The detailed accession numbers of the whole genome sequence data are available in the supplementary Table S2. The whole genome sequence data and annotation data for *C. glauca*, *C. garrawayi*, and *C. inodora* have been deposited in the Genome Warehouse in National Genomics Data Center (Chen et al. 2021; CNCB-NGDC 2022), Beijing Institute of Genomics, Chinese Academy of Sciences/China National Center for Bioinformation, under accession numbers GWHERAX00000000, GWHERAY00000000, GWHERAZ00000000, GWHERBA00000000, GWHERBB00000000, GWHERBC00000000, GWHERBD00000000, GWHERBE00000000, GWHERBF00000000, BioProject [PRJCA022553], and Biosamples [SAMC3299182—SAMC3299184] that are publicly accessible at https://ngdc.cncb.ac.cn/gwh. The whole genome and annotation data of *C. glauca*, *C. garrawayi* and *C. inodora* have also been submitted to Citrus genome database, under the accession numbers CGD24002, CGD24003 and CGD24001 respectively (https://www.citrusgenomedb.org/).
