# Supplementary material for: The genomes of Australian wild limes
Source: Plant Mol Biol. 2024 Sep 24;114(5):102. doi: 10.1007/s11103-024-01502-4 (PMC11422456; doi:10.1007/s11103-024-01502-4)
Supplement: Supplementary file 1 — Supplementary file1 (DOCX 6623 kb) [file 11103_2024_1502_MOESM1_ESM.docx]

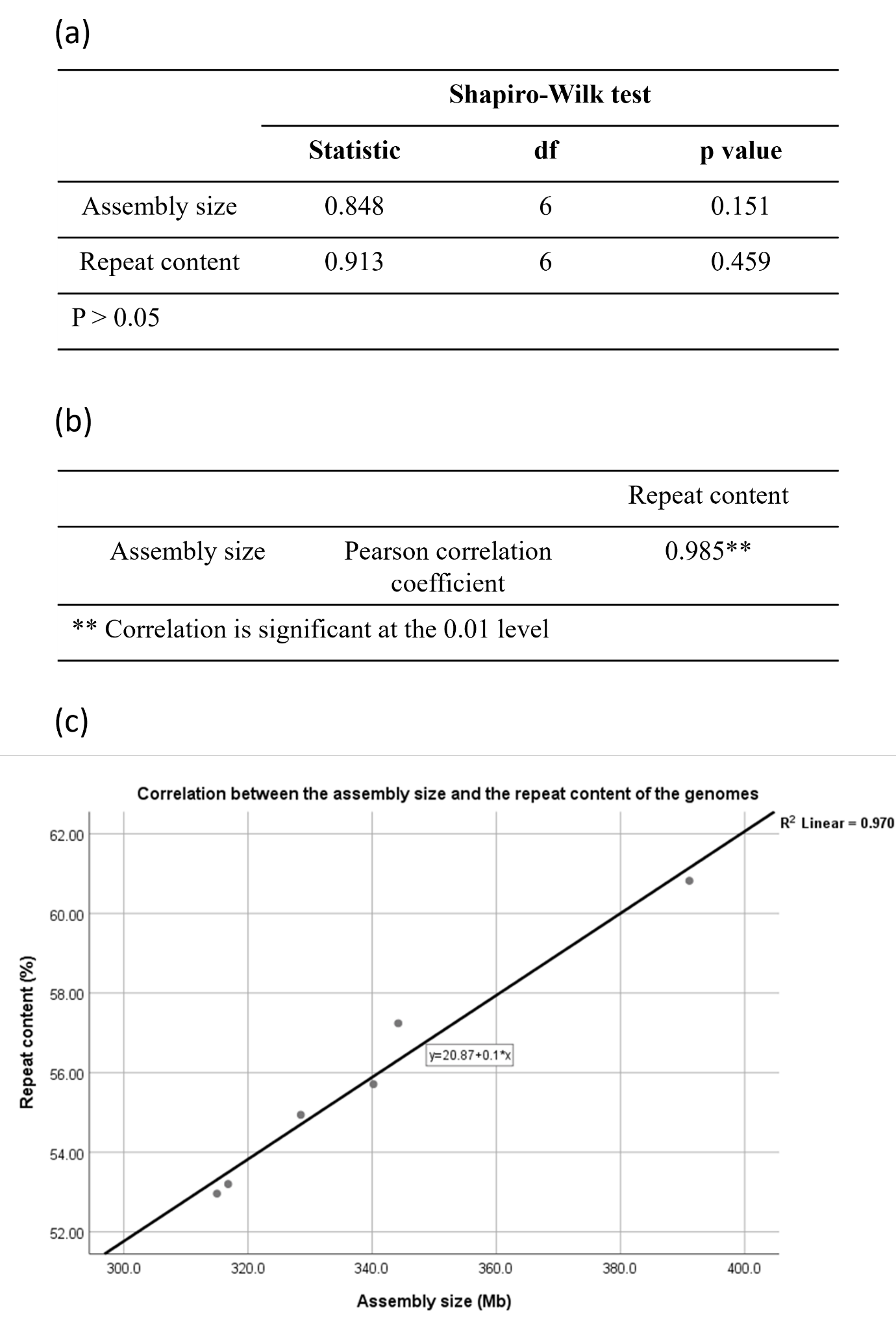


**Figure S1** The correlation between the genome size (Mb) and the total repeat content (%) of the Australian limes.

(a) The data set was found to be normally distributed (p > 0.05) based on the Shapiro-Wilk test. (b and c) There was a strong, positive correlation between the genome size and the repeat content of the genomes (r = 0.985, p < 0.01).


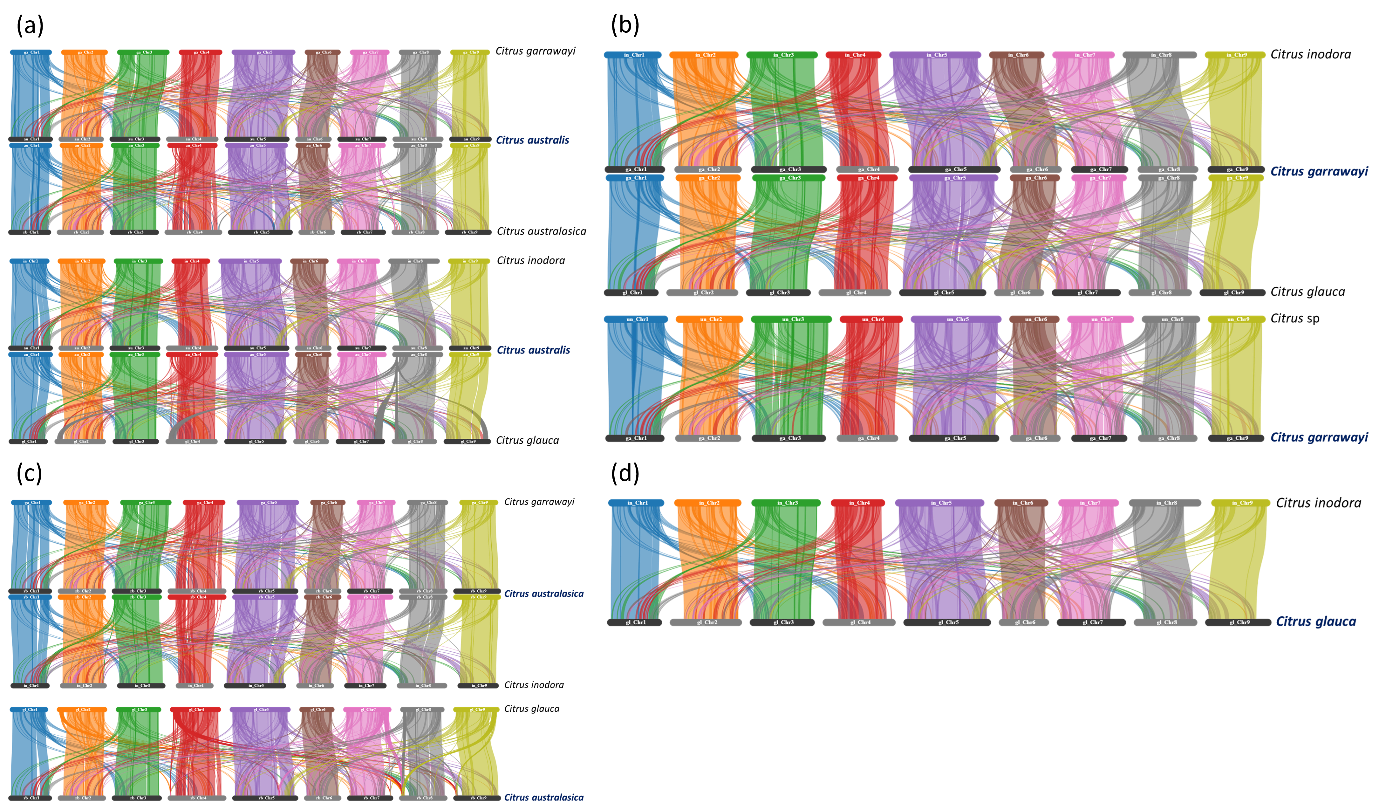


**Figure S2** Collinear genes between chromosomes of all Australian citrus species. (a) Collinear genes found between *C. australis* and other citrus species. (b) Collinear genes found between *C. garrawayi* and other citrus species. (c) Collinear genes found between *C. australasica* and other citrus species. (d) Collinear genes found between *C. inodora* and *C. glauca*.

Most of the collinear genes were found within the same chromosome of any two citrus species. Some genes of Chr1 showed collinearity in Chr3,4,5 and 8 between all the genomes. Similarly, Chr2 of all the species showed collinearity with Chr4,5 and 7. Chr3 had collinear genes in Chr1 and 8. Chr4 had collinear genes with Chr1in all the species and Chr2 in some species. Chr8 showed gene collinearity with Chr3,6,7 and Chr9 showed gene collinearity with Chr3,5,8.


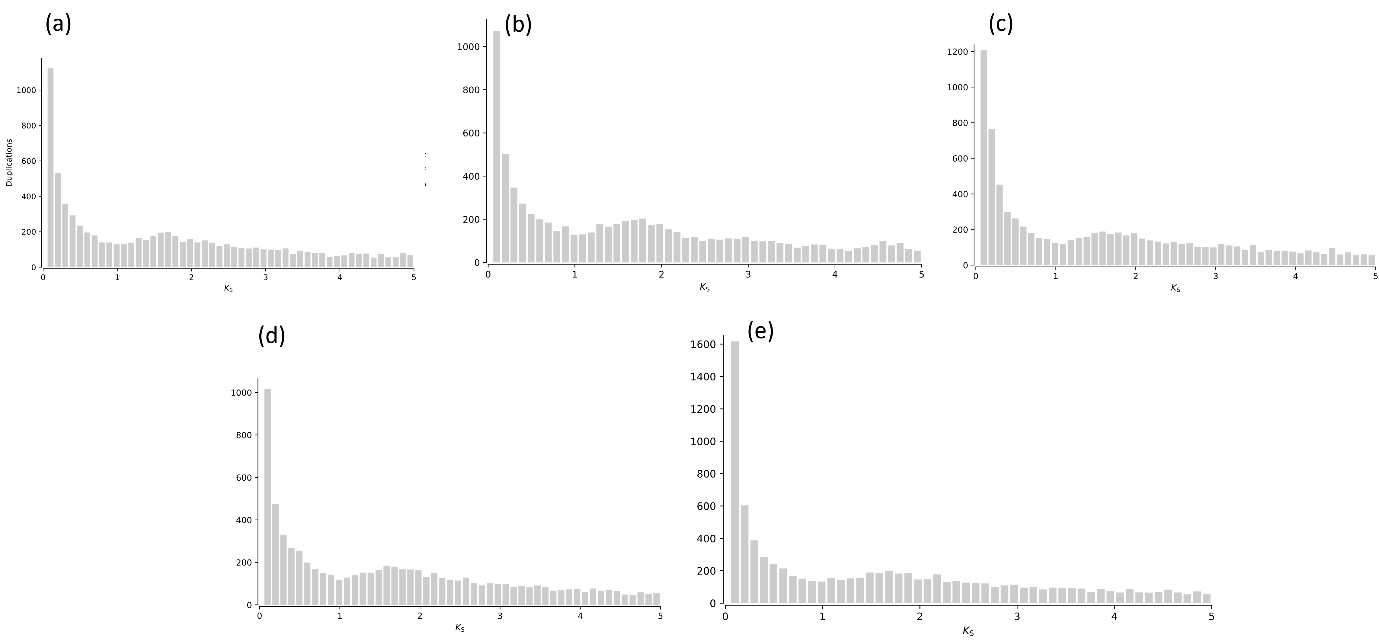


**Figure S3** Ks distribution peaks for paralogous gene pairs of each species. The peaks were generated using WGD tool. (a) *C. australis* (b) *C. garrawayi* (c) *C. glauca* (d) *C. inodora* (e) *C. australasica*. The ks peaks for all species were identified nearly at 1.5.


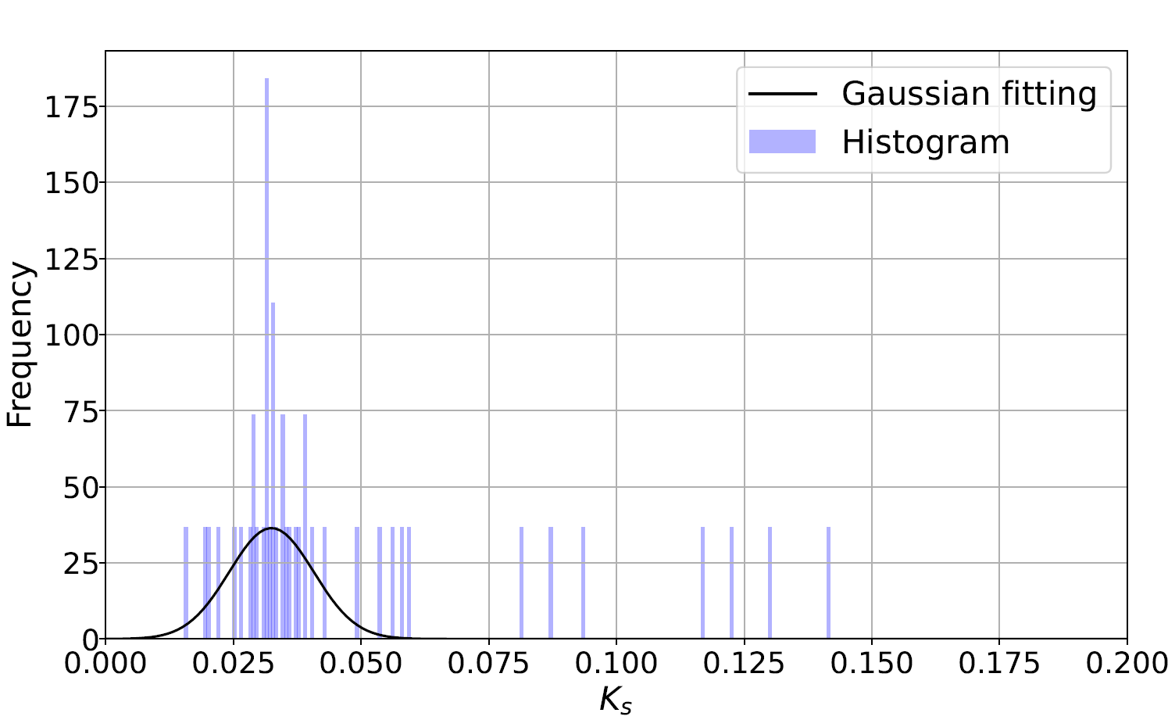


**Figure S4** Ks distribution peak at 0.032 for orthologous gene pairs between *C. australis* and *C. sinensis*. The Ks peak was calculated using WGDI tool.


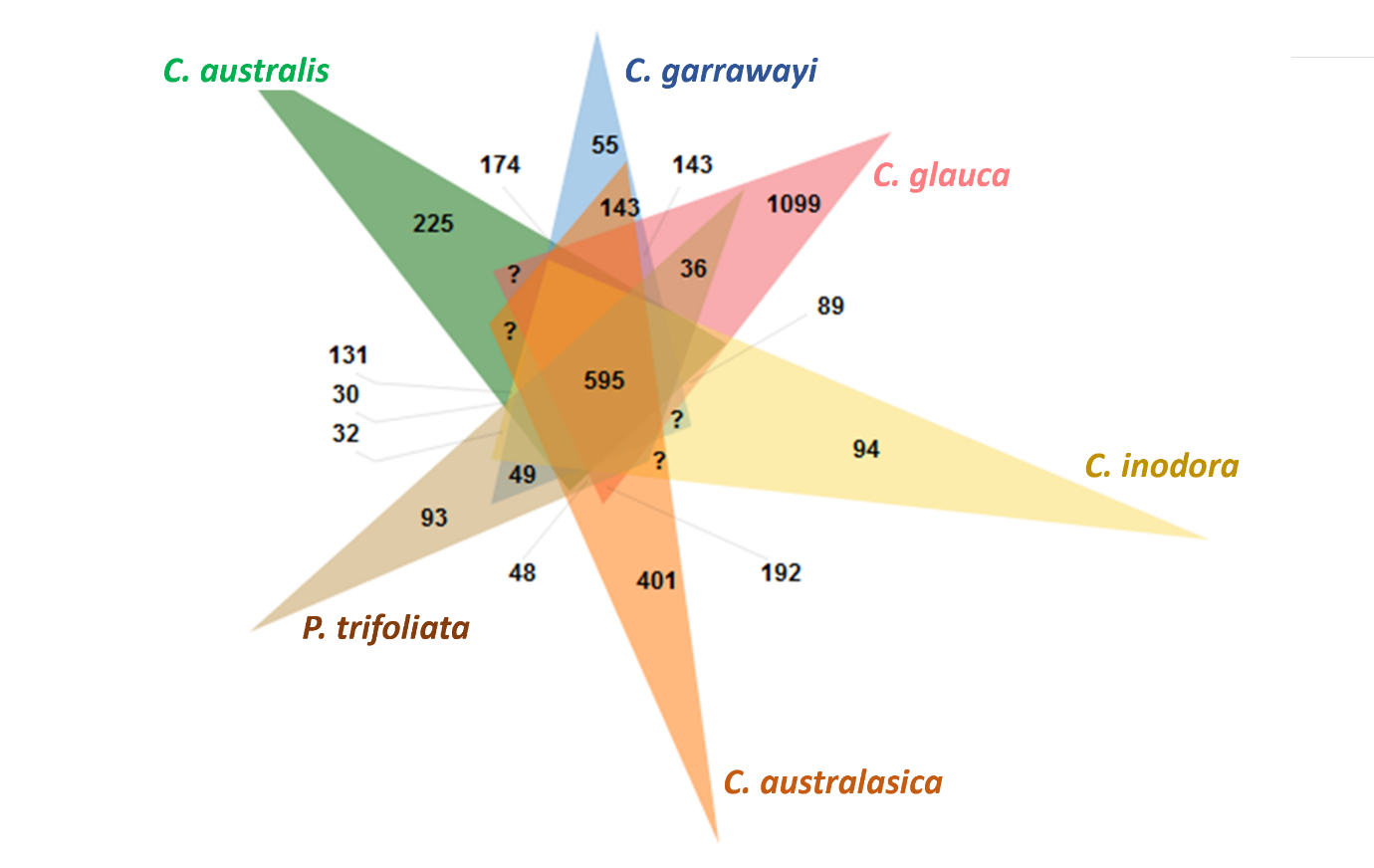


**Figure S5** Venn diagram showing unique and shared orthogroups / gene families among six citrus species including *P. trifoliata* using Orthofinder. All six species shared 595 gene families and *P. trifoliata* had 93 species-specific gene families. The Venn diagram was generated using Orthovenn3.


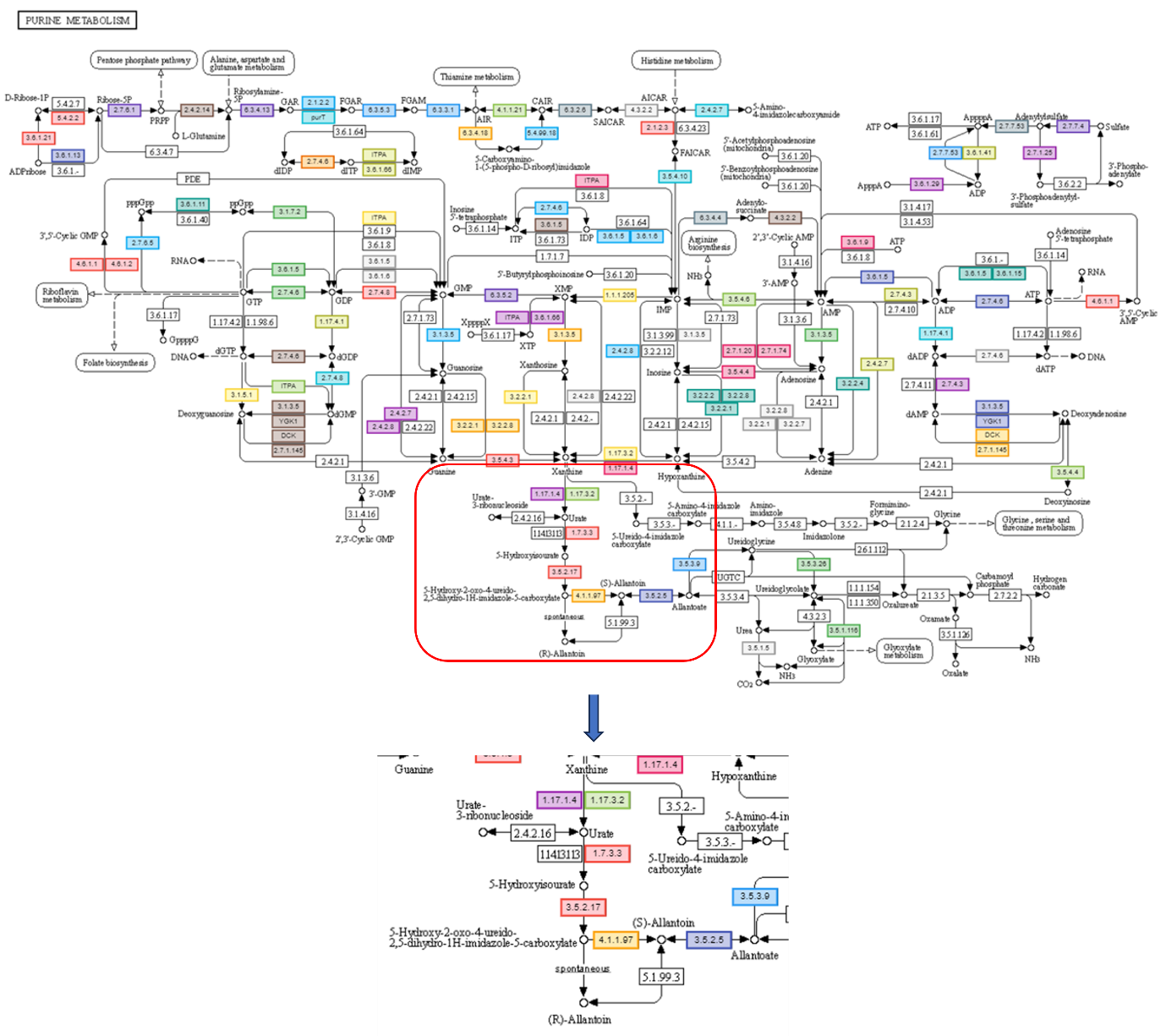


**Figure S6** Purine metabolism pathway. The red coloured box highlights the allantoin synthesis and its conversion to allantoate via allantoinase enzyme (ALN).


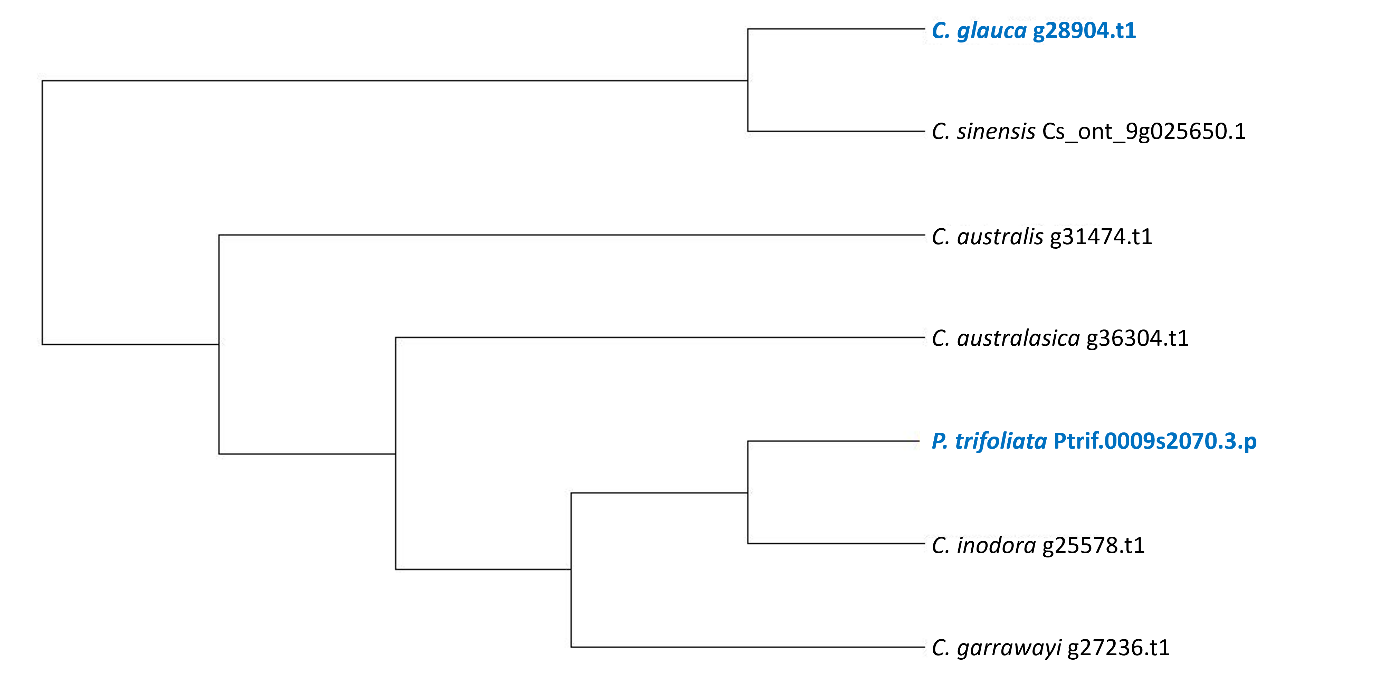


**Figure S7** The gene tree generated using Orthofinder tool, which reflects the orthologous genes among the Australian native citrus, *C. sinensis* and *P. trifoliata*, belonging to the orthogroup OG0009805. Gene tree reveals that two separate events have happened related to this gene family, one shared between *C. glauca* and *C. sinensis*, and another one shared among *C. australis*, *C. australasica*, *P. trifoliata*, *C. inodora*, and *C. garrawayi*. The blue coloured gene of *P. trifoliata* indicates the cold tolerant LOW-TEMPERATURE-INDUCED 65 (LTI65). The homologue of *C. glauca* is also shown in blue colour, which is thought to be involved in cold tolerance.


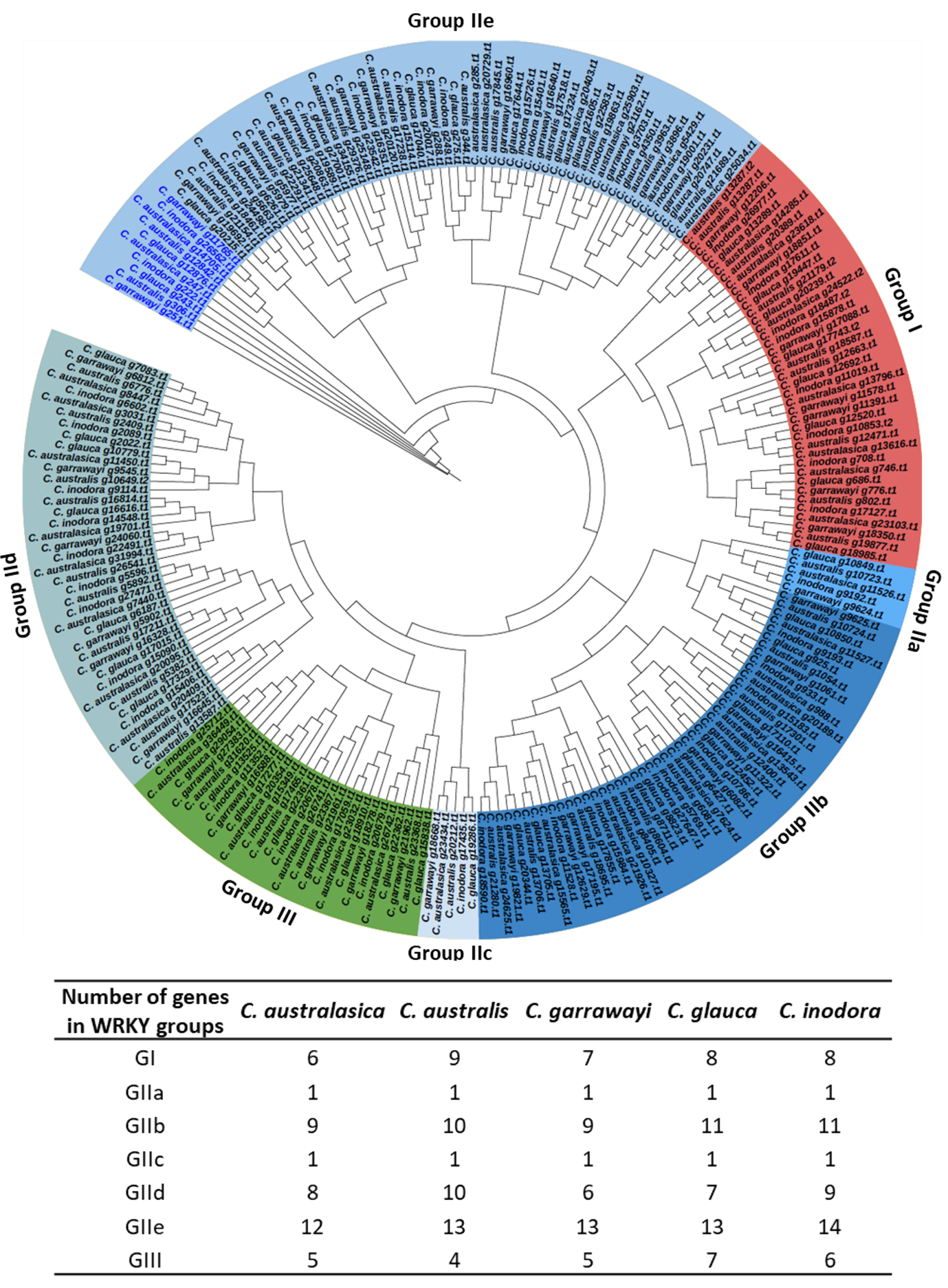


**Figure S8** Maximum Likelihood tree of WRKY domain sequences generated by RAxML method using 1000 bootstrap replicates.
